# Conversation and pragmatics in children who are hard-of-hearing: a scoping review

**DOI:** 10.1093/deafed/enae011

**Published:** 2024-05-16

**Authors:** Jenna Bongioletti, Maree Doble, Alison Purcell

**Affiliations:** Faculty of Medicine and Health, Discipline of Speech Pathology, The University of Sydney, Camperdown, NSW, Australia; Faculty of Medicine and Health, Discipline of Speech Pathology, The University of Sydney, Camperdown, NSW, Australia; School of Health Sciences, Speech Pathology, Western Sydney University, Campbelltown, NSW, Australia

## Abstract

Technological and therapeutic advances have allowed many children who are born hard-of-hearing (HoH) to start school with age-appropriate spoken language skills, yet many of these children continue to find everyday conversations challenging. This scoping review maps the evidence related to development of conversation and pragmatic skills in children who are HoH and learning spoken language. The review followed Arksey and O’Malley’s methodological framework and the PRISMA Extension for Scoping Reviews guidelines. Quality appraisal, data extraction, and thematic analysis were used to describe the data. Systematic searches identified 36 articles for inclusion. Sample sizes were small and heterogenous. Most studies focused on school-aged children with severe hearing loss or greater. Methodological rigor varied. Thematic analysis revealed two global themes. First, children who are HoH continue to find conversation and pragmatics difficult to master, and second, there are a set of audiological, communication, environmental, and demographic characteristics that are associated with better conversation and pragmatic outcomes, some of which are fixed, whereas others are malleable. Focused attention on designing valid and reliable assessments for conversation and pragmatic skills, and on developing therapeutic approaches targeting early conversation and pragmatic skill development, is needed to reduce the impact conversation and pragmatic differences across the lifespan.

## Introduction

Technological and therapeutic advances, and the introduction of newborn hearing screening programs, mean that many children who are born hard-of-hearing (HoH) are now starting formal schooling with age-appropriate spoken language skills as measured by standardized assessments (Ching et al., 2010; Fulcher et al., 2012; Geers et al., 2016; Tobey et al., 2013; Yoshinaga-Itano et al., 2010, 2017; Yoshinaga-Itano & Wiggin, 2016). Opportunities for children who are HoH to develop strong spoken language skills have increased rapidly (Cupples et al., 2018; Dettman et al., 2016; Xie et al., 2014); however, improved spoken language outcomes do not necessarily translate to improved functional conversation and pragmatic abilities. Many children who are HoH and learning spoken language continue to struggle engaging in enjoyable, effective conversations and interactions with their peers (Church et al., 2017; Fitzpatrick et al., 2020; Toe et al., 2007; Toe & Paatsch, 2013). For the purposes of this paper, the term HoH is used to describe a person who has a bilateral hearing loss of any degree (from mild to severe), who communicates or is learning to communicate through spoken language, and who uses hearing aids, cochlear implants (CIs) and/or other assistive listening devices (World Health Organization, 2024).

### Conversation and pragmatics

Conversations are guided by a set of behaviors, known as pragmatics, which include adhering to a set of structured, sequential, verbal, and nonverbal communicative acts (Socher et al., 2019). The aim of successful conversation is to establish and maintain a state of mutual, ongoing understanding (Goodwin & Heritage, 1990). Conversations are a way to create and share meaning, while demonstrating who we are, and what we think, feel, know, and believe (Caissie et al., 1998; Lindsay & Wilkinson, 1999). Our understanding of pragmatic rules, or how to use language appropriately in social contexts, is the foundation on which we build mutual understanding and connect with others (Church et al., 2017; Most et al., 2010).

Conversations involve the use of a range of pragmatic skills, such as taking turns as speaker and listener, introducing topics, making appropriate eye contact, responding contingently, changing topics, and asking questions. When pragmatic expectations are violated, conversational interactions can break down (Clark & Schaefer, 1987, 1989; Goodwin & Heritage, 1990; Sacks et al., 1974; Schegloff, 1992). Breakdowns occur when the flow of the conversation is disrupted, and mutual ongoing understanding is lost (Caissie & Gibson, 1994).

### Conversation and pragmatic skills in children who are HoH

Research exploring the conversation and pragmatic skills in children who are HoH presents conflicting results. Outcomes range from reporting that children who are HoH have significant difficulties with conversation and pragmatic skills, through to finding there were few to no differences between children who are HoH and their typically hearing (TH) peers. These contradictory findings are outlined in a review by Crowe and Dammeyer (2021), which highlighted the variability in pragmatic outcomes of children who are deaf or hard-of-hearing (DHH) and using CI/s in conversational contexts. The review specifically focused on pragmatic skills, in particular speech, acts (six studies), conversational turns (19 studies), and breakdown and repair (three studies), in conversational contexts. Findings reported pragmatic skills of children who are HoH ranging from little to no difficulty, to severe difficulties across each of the pragmatic skills of interest. For example, Toe et al. (2007) reported that the children who are HoH have very few conversational breakdowns and participated in balanced conversations with a familiar teacher, whereas other authors found that children who are HoH spend more time resolving communication breakdowns or in silence (Tye-Murray, 2003), were more likely to experience communication breakdowns (Most et al., 2010; Toe et al., 2007), and were less likely to repair breakdowns successfully (Fitzpatrick et al., 2020) than their TH peers. Furthermore, Crowe and Dammeyer (2021) reported strong positive associations between child characteristics and their pragmatic development including the child’s (a) audiological profile (e.g., use of CIs from an early age, and speech perception skills), (b) speech and language skills (e.g., large vocabulary, strong language skills), (c) the child’s theory of mind, (d) working memory, and (e) gender (being female). This suggests that there may be a range of factors that impact the development of pragmatic and conversational skills for children who are HoH. Identification of possible factors may be a way to improve the pragmatic and conversational skills for all children who are HoH.

In their narrative review, Matthews and Kelly (2022) proposed that participating in spoken conversations is challenging for children who are HoH for a range of reasons, including reduced or inconsistent auditory access because of hearing loss, presence of cognitive or social delays, and/or reduced experience with language provided by fluent language models. However, research examining the natural, unstructured conversations of children who are HoH, suggests that they may not necessarily encounter more, or longer, conversation breakdowns compared with their TH peers, but that they often appear to use different conversation management techniques that set them apart (Church et al., 2017; Fitzpatrick et al., 2020; Toe & Paatsch, 2013). For instance, Toe and Paatsch (2013) found that children who are HoH used a range of pragmatic skills to manage unstructured conversations effectively and avoid breakdowns. They hypothesized that these children asked more questions, made more personal comments, initiated more topics, and took longer turns to stay in control of the conversation and reduce the “risk” of breakdowns occurring (Toe & Paatsch, 2013). By strategically controlling the conversation, children who are HoH and learning spoken language were observed to avoid breakdowns by moving the conversation onto a new topic. Qualitative differences in conversation management were further described by Church et al. (2017), who observed instances of “non-repair” where the child with hearing loss chose not to repair the misspeaking by their TH peer. The absence of repair, where one was needed, resulted in a secondary breakdown which ultimately led to repair. Repair avoidance was not seen at all in the conversations of TH participants.

As mentioned previously, we do not fully understand the necessary factors that contribute to conversation and pragmatic outcomes in children who are HoH. In their narrative review, Matthews and Kelly (2022) concluded that “where present, (pragmatic) delays are consistently explained by the cumulative effects of access to a fluent natural language model” (p. 297), which, in turn, influences the development of language, social, and cognitive skills that are required for successful interactions. It follows that providing children who are HoH with consistent access to fluent natural language models should correlate with improved pragmatic skills; however, this alone does not appear to be sufficient.

### Conversation and pragmatic skills across the lifespan

Consistent access to fluent natural language models occurs most readily when children who are HoH receive hearing healthcare in line with the Joint Committee on Infant Hearing’s “Year 2019 Position Statement: Principles and Guidelines for Early Hearing Detection and Intervention Programs” (Joint Committee on Infant Hearing, 2019). The position statement describes the critical importance of the “1–3–6 benchmark”, that is, hearing screening by 1 month of age, diagnosis by 3 months of age, and accessing intervention by 6 months of age (JCIH, 2019).

The positive influence of technological and therapeutic advances, the introduction of newborn hearing screening and broader adoption of the Joint Standing Committee on Infant Hearing’s 1–3–6 Guidelines (2019) on the spoken language outcomes of children who are HoH have been well documented. Considering this, it seems reasonable to expect that conversation and pragmatic skills have also improved. However, the conflicting results in the evidence base do not confirm that meeting 1–3–6 guidelines alone is sufficient to ensure optimal conversation and pragmatic development. While there are some emerging associations discussed in the literature, we do not fully understand the reasons why some children who are HoH and using spoken language, present with poorer pragmatic and conversational skills compared with their counterparts with TH. As suggested by Goberis et al. (2012), understanding conversation and pragmatic development may be the missing link when it comes to optimizing outcomes for children who are HoH across their lifespans (Xie et al., 2014).

### Toward a better understanding of pragmatic and conversation skill acquisition

A better understanding of the factors that positively influence or negatively impact the acquisition of pragmatic and conversational skills by children who are HoH is needed. One approach includes a biopsychosocial perspective examining the social, psychological, and behavioral dimensions of health and health care, and the interactions between these dimensions (Engel, 1977) using the “Population, Concept, and Context” framework (refer to Table 1). For children with hearing loss, factors requiring consideration include: the type and degree of hearing loss, the age at hearing loss onset, age at diagnosis, cause of hearing loss, the age at which they received hearing technology (e.g., hearing aid/s or CI/s), and whether the device/s in use were an optimal match for the type and degree of hearing loss. Other factors may include the child’s primary mode of communication (spoken, signed, combination), whether the child accessed early intervention supports and from what age, the child’s temperament, level of maternal education, and level of parental engagement in early intervention.

**Table 1:** Defining the Population, Concept, and Concept (PCC) (Pollock et al., 2023).

PCC element	Description
Population	Children from birth to 18 years of age, who are born hard-of-hearing, that is, those with a permanent bilateral hearing loss present from birth. Children who are using hearing aid/s and/or (a) cochlear implant/s and who are learning any spoken language (not sign language). Children without other developmental considerations that could impact on communication development.
Concept	Novel research reporting on any aspect of conversation or pragmatic skills in the population of interest.
Context	Research from any country and reporting on participants speaking any language.

The factors outlined above influence a child’s developmental trajectory, but not all are changeable. Some factors are fixed and cannot be altered (e.g., degree of hearing loss, mothers’ level of education), and others are malleable (e.g., how much an individual uses their hearing technology, and the quantity and quality of language they are exposed to, time in intervention) (McCreery et al., 2015; Moeller et al., 2009; Nailand et al., 2021; Walker et al., 2013). Each child has a unique set of factors that facilitate conversation and pragmatic development in the literature, of fixed and malleable factors related to conversation and pragmatic mastery in this population. Defining fixed and malleable factors that influencing pragmatic and conversational skill development of children who are HoH is an important step toward mitigating the impacts of prelinguistic hearing loss on pragmatic and conversational skill development.

### Scoping review questions

To further explore the pragmatic and conversational abilities of children who are HoH and learning spoken language, and to discover the fixed and malleable factors that may impact them, a scoping review was conducted. A scoping review was selected as the preferred approach to map the literature in this evolving area and identify gaps in the literature (Munn et al., 2018). This review is unique in that it considers children with any degree of bilateral hearing loss, and who are using any type of hearing technology. Recent reviews have considered children using CIs rather than hearing aids and therefore, tend to present the evidence as it relates to children with more significant degrees of hearing loss. This review adopted a broad definition of “conversation” and “pragmatics” considering all research that related to either area without constraining the search to a specific area or areas of conversation or pragmatic development.

The primary scoping review question asked was, “How do children who are HoH from birth, and who use hearing aids and/or cochlear implants to learn a spoken language, develop conversation and pragmatic skills?”. The secondary scoping review question asked was, “Are there factors, fixed or malleable, that correlate with improved conversation and pragmatic outcomes in children who are HoH from birth?”

## Methods

This scoping review was conducted following the methodological framework originally proposed by Arksey and O'Malley (2005) and elaborated on by Levac et al. (2010) and Peters et al. (2015). A scoping review was selected as the appropriate approach to summarizing the evidence base because the intention of the review was to identify knowledge gaps in the evidence base, which the authors anticipated would be relatively small and heterogenous (Munn et al., 2018). The review has been reported in accordance with the PRISMA Extension for Scoping Reviews (PRISMA-ScR) Checklist (Tricco et al., 2018). Search terms, including both Medical Subject Headings (Ganesh et al., 2022) and non-MeSH terms, key words, and truncations, were developed and tested using the logic grid technique described by Aromataris and Riitano (2014) and are summarized in Table 2. The scoping review was pre-registered on the Open Science Framework with details of the scoping review made publicly available prior to commencing the review (https://osf.io/b5tvh/?view_only=545b4e86d43e49d3974647d592b35a7d).

**Table 2:** Search terms.

Key words	Alternative words
Child^*^.mp.	OR p?ediatric^*^.mp. OR congenit^*^.mp. OR Infant^*^.mp. OR adolescent.mp. OR school age.mp. OR preschool.mp. OR preschool age^*^.mp.
AND	
Hearing loss^*^	OR hearing impair^*^.mp. OR deaf.mp. OR hard of hearing.mp.
AND	
Communicat^*^	listen and speak.mp. OR talk^*^.mp. OR speak^*^.mp. OR oral.mp. OR oral language.mp. OR speech.mp. OR spoken language.mp. OR language^*^.mp.
AND	
Hearing aid^*^.mp.	OR hearing device^*^.mp. OR cochlear implant^*^.mp. OR “hearing technology”.mp.
AND	
Convers^*^.mp.	OR pragmatic^*^.mp.

A combination of subject specific and multidisciplinary databases was selected to cover depth and breadth of coverage in the literature search. The initial search strategy was designed, tested, and executed in Medline via Ovid (See Appendix 1), before the search was translated for use with additional databases which included: Embase via Ovid SP (1946–2022), Eric via Ovid SP (1966–2022), CINAHL via EBSCO (1982–2022), and Scopus (2004–2022). Reference lists from studies published in the last five years were also hand searched for completeness. All searches were conducted on April 12, 2022 and results from each search were exported into EndNote X9 for reference management, and into Covidence, a web-based collaboration software platform that streamlines the production of systematic and other literature reviews (www.covidence.com.au).

Articles were included if they presented novel, peer-reviewed research that was written in English and published between January 2000 and April 2022. Articles had to focus on the conversation and pragmatic skills of children aged 0 to 18 years, who are HoH and learning spoken language (rather than sign language). HoH participants had to have a permanent bilateral hearing loss, or any degree, that was present from birth. HoH participants also had to be using hearing technology (hearing aid/s or CI/s) and have no other developmental concerns impacting on their communication skills. Because of the small number of studies that were anticipated to meet inclusion criteria, papers using quantitative, qualitative, and mixed methodologies were considered.

Automatic de-duplication in Covidence and manual checks were used to verify and remove duplicate records (Bramer et al., 2016). All remaining records were screened by authors 1 and 3, initially by title and abstract, and then full texts were assessed against a-priori inclusion criteria which are listed above. Where decisions were conflicting, both authors reread the entire article and engaged in discussion of their perspective on the fit of the study to the research question until 100% consensus agreement was reached (Li et al., 2022).

While Arksey and O'Malley (2005) stated that quality appraisal is not required in scoping reviews, Tricco et al. (2018) suggest quality appraisal as a useful addition to improve methodological rigor. Therefore, authors 1 and 2 completed quality appraisal on 20% of the included articles using the Joanna Briggs Institute (JBI) (2017) checklist for analytical cross-sectional studies (see Appendix 2). Initial inter-rater reliability was 72%, individual perspectives were discussed until 100% consensus agreement was reached (Li et al., 2022). Once completed, key information was extracted as presented in Appendix 3 (studies on conversation) and Appendix 4 (studies on pragmatics) Appendices 2. Extracted information included details of the article (title, authors, year, country, participant language), objective/s or research question/s, participant characteristics and context, and relevant study outcomes.

Finally, inductive thematic analysis was conducted following Braun and Clarke’s (2006) six-phase approach. In the first phase, the first author reread each article and noted down key findings that related to the research question. In the second phrase, author 1 annotated the articles to identify key themes that were coded and recorded against each article in an excel spreadsheet. In phase 3, coded themes were transferred to Jamboard (www.jamboard.google.com), a digital whiteboard for real-time virtual collaboration, before three authors reviewed the codes and grouped ideas into global themes which were then discussed and refined collaboratively. Phase 4 saw the authors consider and collaboratively code for subthemes within each of the global themes. Subthemes were further categorized as either fixed or malleable where this was relevant. In phase 5, the themes and subthemes were revised and further defined to make sure that the key concepts from the data were appropriately captured. Phase 6 saw the authors writing up the findings of the thematic analysis and relating this back to the predetermined review questions.

## Results

**Figure 1:**
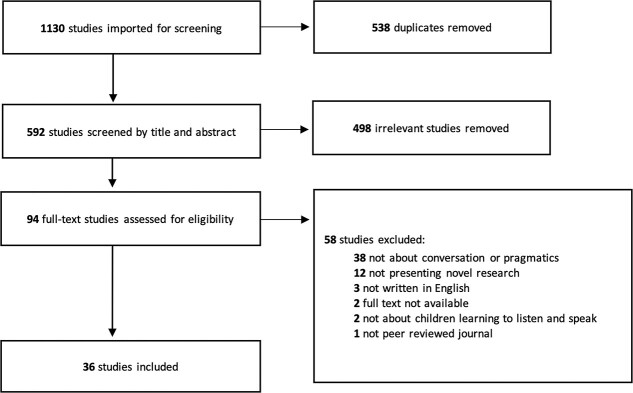
PRISMA flow diagram.

Systematic searching yielded 1,130 citations, and 538 duplicate references were immediately removed. Title and abstract screening by two authors identified a further 298 articles as irrelevant. The remaining 94 articles were read by two authors at the full-text screening level, with 36 articles ultimately meeting the inclusion criteria as displayed in the Preferred Reporting Items for Systematic Reviews and Meta-Analysis (Moher et al., 2015) flow diagram in Figure 1.

There were 34 studies that reported on non-randomized observational cross-sectional data, and two studies presenting longitudinal data collected over periods of up to 3 years. More studies focused on measuring conversation skills (*n* = 21), than on measuring pragmatic skills (*n* = 15). Samples sizes ranged from 3 to 126 HoH participants (mean = 28.8, *SD* = 29.0). There was heterogeneity within samples, with a variation in factors including participant age, degree of hearing loss, age at diagnosis, age at fitting of hearing device/s, type of hearing device/s used, access to early intervention. Nearly half the studies included examined outcomes of primary school-aged participants only (*n* = 15), with a further 6 studies included some primary school-aged students as part of a broader range of participant ages. There were another six studies that reported on skills in high school children, meaning that well over half the studies (*n* = 21) studies focused exclusively on school-aged children, including both primary school-aged children from ~5;0 to 11;11 years old and high school-aged children from ~12;0 to 17;11 years old. In comparison, just six studies reported specifically on skills of children in their early childhood years (children from birth to 3;11 years and who have not yet started formal preschool program, which occurs at around 4;0 years) or in their preschool years (children between 4;0 and 4;11 years who are participating in a preschool program). Another four studies reported on the skills of some younger children as part of a broader cohort of primary and high school-aged children.

Over half the studies (*n* = 25) included participants with severe and profound hearing loss only. The remaining studies (*n* = 11) included children with hearing losses ranging from mild to profound, with children who had a mild or moderate hearing loss underrepresented in the data. In total, 20 of the 36 studies were conducted in English-speaking countries (Australia, United States, Canada, United Kingdom), with the remaining 16 studies originating in countries where English is not the dominant language. These countries (and languages spoken) included Sweden (Swedish), Italy (Italian), Israel (Hebrew), Egypt (Arabic), France (French), and Iran (Persian).

### Studies: conversation skills

Of the 21 studies measuring conversational outcomes, 13 reported on skills observed or reported on during free conversation, and eight reported on skills observed in referential communication tasks. A range of conversational phenomena were measured using a range of tools and techniques, including measurement of the number of conversation breakdowns/requests for clarification, duration (in turns) of conversation breakdowns, types of repair requests, proportion of repair requests of each type, responses to repair requests, types of repairs used, proportion of turns taken by each participant (conversational balance), length of conversational turns (in words), number of questions versus statements used, number of topics per conversation, number of turns per topic (topic maintenance), number of speech acts by category (e.g., assertive, directives, expressives/conversational turn types), and social competence (measures of assertiveness, egocentrism, responsiveness, awareness, affiliation, reciprocity, mutuality, and social problem-solving skills).

### Studies: pragmatic skills

Of the studies measuring pragmatic skills (*n* = 15), 11 used observational rating scales and the remaining four used standardized pragmatics assessments. The range of standardized and nonstandard tools used to measure pragmatic skills is summarized in Table 3. Studies focused predominantly on pragmatic skill development in primary and high school-aged participants. Studies included both hearing aid users and CI users, most of whom were children with severe hearing loss or greater. Again, there was substantial variability in participant profiles, and there was little consistency in collection of and reporting on key confounding variables.

**Table 3:** Standardized and nonstandard measures of pragmatic skills.

Standardized measure used	Number of uses
Egyptian Arabic Pragmatic Language Test (EAPLT) (Khodeir et al., 2017)	2
Pragmatic Language Skills Test (APL MEDEA) + Colors Game (Lorusso, 2009)	2
Le Abilità Socio-Conversazionali del Bambino (Girolametto, 1997)	1
**Nonstandard measure used**	**Number of uses**
The Pragmatics Checklist (Goberis et al., 2012)	2
The Pragmatics Protocol (Prutting & Kirchner, 1987)	1
Pragmatics Profile from the Clinical Evaluation of Language Fundamentals (4th edition) (CELF-4) (Semel et al., 2003)	2
Children’s Communication Checklist (2nd edition) (CCC-2) (Bishop, 2003)	2
Strengths and Difficulties Questionnaire (SDQ) (Goodman, 1997)	1
Self-developed checklists (authors wrote and used own checklist)	5

### Results of quality appraisal

In accordance with the JBI Levels of Evidence guidelines (Joanna Briggs Institute, 2013), included studies were categorized as level 4b evidence, that is, observational-descriptive studies. This level of evidence is considered low quality (Joanna Briggs Institute, 2013; Petrisor & Bhandari, 2007). Studies presented either non-randomized cross-sectional data (*n* = 34) or longitudinal data (*n* = 2) collected over periods of up to 3 years. Studies included quantitative, qualitative, and mixed methodologies. The methodological quality of studies varied. Inclusion criteria were usually not determined a-priori, with only two studies clearly identifying predetermined inclusion criteria. All studies reported on participant characteristics in some form; however, there was little consistency in terms of the characteristics that measured and reported on. Similarly, the extent to which confounding variables were identified, measured, and analyzed varied between studies. Validity and reliability were inconsistently addressed in the research. Results of the quality appraisal are included in Appendix 1.

### Thematic analysis

**Figure 2:**
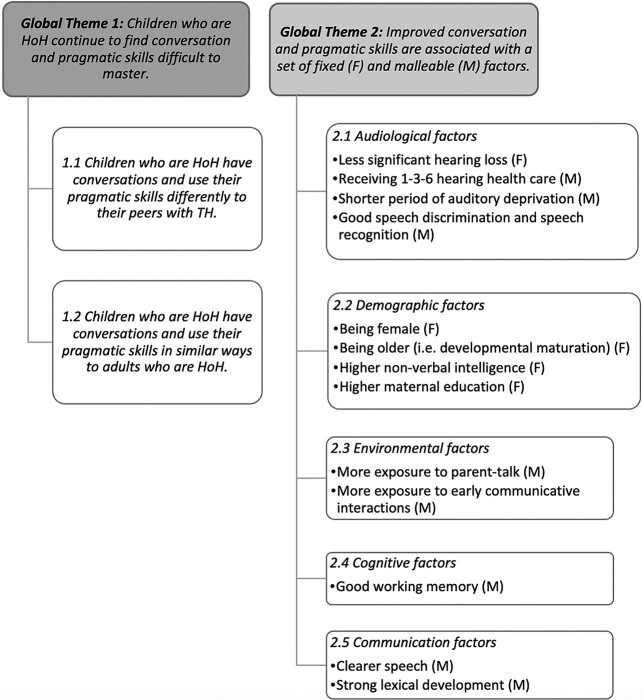
Global themes and subthemes.

Thematic analysis of the scoping review revealed two themes, as outlined in Figure 2.

In summary, the first theme highlights that children who are HoH continue to find conversation and pragmatics difficult to master. Their skills are different to those of their peers with TH, and difficulties appear to persist into adulthood with conversation and pragmatic behaviors observed in childhood echoing behaviors observed in adults who are HoH. The second theme identifies a set of audiological, communication, environmental, and demographic characteristics that are associated with better conversation and pragmatic outcomes in children who are HoH. Some of these factors are fixed (F), whereas others are malleable (M).

## Discussion

The aims of this scoping review were to (a) understand how children who are HoH from birth, and who use hearing aids and/or CIs to learn a spoken language, develop conversation and pragmatic skills, and (ii) to identify factors, fixed or malleable, that correlated with improved conversation and pragmatic outcomes in the population of interest. The findings highlight that children who are HoH from birth encounter differences in the development of conversation and pragmatic skills to their peers with TH. In addition, there are a set of fixed and malleable factors that may improve the pragmatic and conversational skills of children who are HoH from birth. These findings need to be considered within the context that the number of studies that met inclusion criteria was small, and within these studies, cohorts were small and heterogenous. Inconsistent reporting on participant characteristics (i.e., degree of hearing loss, type hearing technology used, age at diagnosis, age at amplification) also made generalization of results challenging. Further to this, researchers did not agree on which conversation and pragmatic phenomena warranted assessment and observation, with a broad range of assessment measures, quantifying and qualifying a broad spectrum of skills, and often lacking validity and reliability. The outcome of this is that findings have not been able to be replicated, nor can findings be reported with confidence. Finally, the research rigor of the included studies was low with all studies rated as level 4b evidence, that is, observational-descriptive studies (Joanna Briggs Institute, 2013).

### Children who are HoH continue to find conversation and pragmatic skills difficult to master

The thematic analysis (Braun & Clarke, 2006) affirmed that children who are HoH continue to find conversation and pragmatic skills difficult to master. While this has been evident in the literature for some time (Church et al., 2017; Goberis et al., 2012; Hilviu et al., 2021; Most et al., 2010; Paatsch & Toe, 2014; Remine et al., 2003; Rinaldi et al., 2013; Shoeib et al., 2016; Zaidman-Zait & Most, 2020), it is perhaps surprising, given the advances in the oral language skills of children who are HoH in recent years. The data suggest that conversational and pragmatic skills do not correlate to the child’s level of language mastery, as even children with age-appropriate spoken language skills do not necessarily have age-appropriate conversation and pragmatic skill development (Dammeyer, 2013; Rinaldi et al., 2013).

One possible explanation for the difference between language mastery, and conversational skill development, could relate to findings that children who are HoH, and learning spoken language, relying on a good communication partner to support these skills. Lloyd et al. (2001) found that children who are HoH took less turns when talking with teachers, whereas teachers took more and longer turns. Similarly, Toe et al. (2007) found that children who are HoH and learning spoken language tend to do better in conversation with teachers of the deaf (ToD), encountering less breakdowns and improved conversational balance with their ToD. The authors suggested that the way the ToD communicated with children was more facilitative of successful interactions. Many children who are HoH, and learning spoken language, spend a lot of time in intervention, interacting with adults from very early in their development, where children are set up for success in conversations. Parents and teachers appear to adjust their talk to facilitate improved understanding, thereby minimizing the need for conversation repair. It may be that in our attempts to support and enhance language development, we are inadvertently sabotaging opportunities for children who are HoH to practice identifying and resolving breakdowns.

While advances made over the past two decades have resulted in better spoken language outcomes for children who are HoH, progress toward routinely meeting the 1–3–6 guidelines varies from country to country which may explain some of the variation in the data. Further to this, the number of studies that met inclusion criteria was small, and within these studies, cohorts were small and heterogenous. Inconsistent reporting on participant characteristics (i.e., degree of hearing loss, type hearing technology used, age at diagnosis, age at amplification) also made generalization of results challenging. Similar issues are often seen in the broader evidence base relating to children who are HoH generally. Crowe and Dammeyer (2021) observed that methodological issues in the literature made it difficult to distill a clear picture of conversational and pragmatic outcomes in CI users, and this review corroborates their finding and extends the statement to apply to the broader population of children who are HoH and using both HAs and CIs.

In the absence of targeted interventions, conversation and pragmatic difficulties appear to persist into adulthood, with children and adolescents who are HoH observed behaving in similar ways to adults who are HoH. Both children and adults who are HoH have been found to take longer conversational turns, introduce more topics, take less turns per topic and encounter more and longer conversational breakdowns that are resolved less efficiently (Paatsch & Toe, 2014; Toe & Paatsch, 2013).

While these differences may seem benign at first glance, over time, adults who are HoH tend to begin avoiding situations where breakdowns are more likely to occur (Stephens et al., 1999) and are ultimately at risk for experiencing difficulties across areas including social and emotional well-being, educational attainment, and employment (Holzinger & Fellinger, 2022; Erber, 2002; Heine & Browning, 2004). Future research should consider conversation and pragmatic development in the early years where targeted interventions may have the most impact.

### Children who are HoH continue to need support with conversation and pragmatic development

The second theme identified that better conversation and pragmatic skills are associated with audiological, demographic, environmental, communication, and cognitive factors (refer to Figure 2). Categorizing these factors as either fixed or malleable is a unique approach to describing these data that may yield insight into how best to target improved conversation and pragmatic skills in the population of interest. While fixed factors cannot be altered by intervention, they may help us identify children’s risk for conversation and pragmatic difficulties for close monitoring and priority access to intervention.

Of the malleable factors identified, several are already known to positively influence communication development. For example, there is a well-established relationship between receiving 1–3–6 hearing health care and improved speech discrimination, clearer speech production, better working memory, and better lexical development (Ambrose et al., 2014; Ching et al., 2010; Geers et al., 2009; Tomblin et al., 2014, 2020; Yoshinaga-Itano et al., 2010). There is also an established relationship between improved language outcomes, and the degree of engagement in early communicative interactions (Hart & Risley, 1995). What is not yet understood is what types of parent-talk and early communicative interaction led to better conversation and pragmatic outcomes in children who are HoH. Increased focus on understanding how malleable factors can facilitate improved outcomes, may offer a starting point for the development of novel intervention approaches targeting conversation and pragmatic development in the children who are HoH.

It is possible that there are other factors that positively impact the development of pragmatics and conversational skills that are not evident at this stage. Researchers and clinicians need to improve assessment of, and develop interventions for, pragmatic and conversation skills; particularly as improvements in listening and spoken language skills do not appear to result in equivalent improvements in conversation and pragmatic skills (Dammeyer, 2013; DeLuzio & Girolametto, 2011; Rinaldi et al., 2013).

### Preliminary recommendations for clinicians

While there is variation in the conversation and pragmatic outcomes of children who are HoH, there appears to be some factors associated with conversation and pragmatic success. The following preliminary recommendations for clinical practice are proposed as a useful starting point for clinicians. As these have been collated from a small sample of studies, they should be viewed with caution. To assist with the identified malleable factors service providers can provide intervention that:

1. Promotes early diagnosis, early and appropriate fitting of hearing technology, and timely entry into early intervention in line with the Joint Committee of Infant Hearing’s 1–3–6 guidelines to minimize the duration of auditory deprivation.
2. Develops strong speech perception and speech recognition skills. By doing so, children develop a strong auditory feedback loop which will allow them to hear conversation around them.
3. Develops clear and intelligible speech skills so they can be more easily understood by others.
4. Guides and coaches parents to provide children strong language models with lots of exposure to parent talk and opportunities to engage in early communicative interactions.
5. Includes regular assessment of conversation and pragmatic skills for all children who are HoH from early childhood years onwards, ideally using standardized measures that can inform intervention.

Furthermore, service providers may improve the development of pragmatic and conversational skills of children who are HoH by providing intervention designed specifically for each of the unique set of fixed factors that come with each child and their family. For instance, the development of pragmatic and conversational skills could be incorporated into intervention sessions more frequently with children with greater degrees of hearing loss, who are younger, or for families with lower levels of maternal education.

## Conclusion

Children who are HoH and learning spoken language, continue to have difficulty mastering conversation and pragmatic skills that form the foundation for relationships with others. Children who are HoH display conversation and pragmatic skills that are different to their peers with TH, even in the presence of age-appropriate spoken language skills. Children who are HoH use conversation and pragmatic behaviors that often mirror those observed in adults who are HoH. This suggests that conversation and pragmatic difficulties continue into adulthood in the absence of targeted intervention. Improved conversation and pragmatic skills may be associated with a set of audiological, demographic, environmental, cognitive, and communication factors that can be categorized as either fixed or malleable. These findings should be interpreted with caution because of the small number of available studies that present low-level evidence. Future research should focus on developing an in-depth understanding of how conversation and pragmatic skills of DHH children develop in early childhood, using study designs that add depth, confidence, and generalisability to the knowledge base. Developing interventions that target conversation and pragmatic skill development in early childhood, alongside traditional intervention for development of spoken language skills, will be a priority if we aim to reduce or avoid the impact of conversation and pragmatic difficulties across the lifespan.

## Funding

Authors received no financial support for this research.

##  

*Conflicts of interest:* None declared.

## Supplementary Material

Appendix_1_enae011

Appendix_2_enae011

Appendix_3_enae011

Appendix_4_enae011

All_appendices-Revised_enae011

## References

[ref1] Alduais, A. (2012). Use of an Arabic-language version of TOPL-2 to identify typical and atypical manifestations of pragmatic language impairment in individuals with developmental dysphasia. IOSR Journal of Humanities and Social Science, 3, 11–22. 10.9790/0837-0331122

[ref2] Ambrose, S. E., Unflat Berry, L. M., Walker, E. A., Harrison, M., Oleson, J., & Moeller, M. P. (2014). Speech sound production in 2-year-olds who are hard of hearing. American Journal of Speech Language Pathology, 23(2), 91–104. 10.1044/2014_AJSLP-13-0039 24686852 PMC4035418

[ref3] Arksey, H., & O'Malley, L. (2005). Scoping studies: Towards a methodological framework. International Journal of Social Research Methodology, 8(1), 19–32. 10.1080/1364557032000119616

[ref4] Aromataris, E., & Riitano, D. (2014). Constructing a search strategy and searching for evidence. A guide to the literature search for a systematic review. American Journal of Nursing, 114(5), 49–56. 10.1097/01.NAJ.0000446779.99522.f6 24759479

[ref5] Bishop, D. (2003). Children’s communication checklist (CCC-2). In: Volkmar, F.R. (Eds.). Encyclopedia of Autism Spectrum Disorders. Springer, New York, NY. 10.1007/978-1-4419-1698-3_1929

[ref6] Bramer, W. M., Giustini, D., de Jonge, G. B., Holland, L., & Bekhuis, T. (2016). De-duplication of database search results for systematic reviews in EndNote. Journal of the Medical Library Association, 104(3), 240–243. 10.3163/1536-5050.104.3.014 27366130 PMC4915647

[ref7] Braun, V., & Clarke, V. (2006). Using thematic analysis in psychology. Qualitative Research in Psychology, 3(2), 77–101. 10.1191/1478088706qp063oa

[ref8] Caissie, R., Dawe, A. L., Donovan, C., Brooks, H., & MacDonald, S. M. (1998). Conversational performance of adults with a hearing loss. Journal of the Academy of Rehabilitative Audiology, 31, 31–44.

[ref9] Caissie, R., & Gibson, C. L. (1994). The effectiveness of repair strategy intervention with a hearing-impaired adult. Journal of Speech-Language Pathology and Audiology, 18(1), 14–22.

[ref10] Ching, T. Y., Crowe, K., Martin, V., Day, J., Mahler, N., Youn, S., Street, L., Cook, C., & Orsini, J. (2010). Language development and everyday functioning of children with hearing loss assessed at 3 years of age. International Journal of Speech-Language Pathology, 12(2), 124–131. 10.3109/17549500903577022 20420353 PMC3094718

[ref11] Church, A., Paatsch, L., & Toe, D. (2017). Some trouble with repair: Conversations between children with cochlear implants and hearing peers. Discourse Studies, 19(1), 49–68. 10.1177/1461445616683592

[ref12] Clark, H., & Schaefer, E. (1987). Collaborating on contributions to conversations. Language and Cognitive Processes, 2(1), 19–41. 10.1080/01690968708406350

[ref13] Clark, H. H., & Schaefer, E. F. (1989). Contributing to discourse. Cognitive Science, 13(2), 259–294. 10.1207/s15516709cog1302_7

[ref14] Crowe, K., & Dammeyer, J. (2021). A review of the conversational pragmatic skills of children with cochlear implants. Journal of Deaf Studies and Deaf Education, 26(2), 171–186. 10.1093/deafed/enab001 33740059

[ref15] Cupples, L., Ching, T. Y., Button, L., Seeto, M., Zhang, V., Whitfield, J., Gunnourie, M., Martin, L., & Marnane, V. (2018). Spoken language and everyday functioning in 5-year-old children using hearing aids or cochlear implants. International Journal of Audiology, 57(sup2), S55–S69. 10.1080/14992027.2017.1370140 PMC5847428 28899200

[ref16] Dammeyer, J. (2013). A longitudinal study of pragmatic language development in three children with Cochlear implants. Deafness & Education International, 14(4), 217–232. 10.1179/1464315412z.00000000024

[ref17] DeLuzio, J., & Girolametto, L. (2011). Peer interactions of preschool children with and without hearing loss. Journal of Speech Language & Hearing Research, 54(4), 1197–1210. 10.1044/1092-4388(2010/10-0099) 21173389

[ref18] Dettman, S. J., Dowell, R. C., Choo, D., Arnott, W., Abrahams, Y., Davis, A., Dornan, D., Leigh, J., Constantinescu, G., Cowan, R., & Briggs, R. J. (2016). Long-term communication outcomes for children receiving cochlear implants younger than 12 months: A multicenter study. Otology & Neurotology, 37, e82–e95. 10.1097/MAO.0000000000000915 26756160

[ref19] Engel, G. L. (1977). The need for a new medical model: A challenge for biomedicine. Science, 196(4286), 129–136. 10.1126/science.847460 847460

[ref20] Erber, N. P. (2002). Hearing vision communication and older people (Vol. 23, pp. 035–042). Clavis.

[ref21] Fitzpatrick, E., Squires, B., & Kay-Raining Bird, E. (2020). What's that you say? Communication breakdowns and their repairs in children who are deaf or hard of hearing. Journal of Deaf Studies and Deaf Education, 25(4), 490–504. 10.1093/deafed/enaa010 32463866

[ref22] Fulcher, A., Purcell, A. A., Baker, E., & Munro, N. (2012). Listen up: Children with early identified hearing loss achieve age-appropriate speech/language outcomes by 3 years-of-age. International Journal of Pediatric Otorhinolaryngology, 76(12), 1785–1794. 10.1016/j.ijporl.2012.09.001 23084781

[ref23] Ganesh, B. B., Damodar, B. V., Dharmesh, R., Karthik, K. R. T., & Vasudevan, S. K. (2022). An innovative hearing-impaired assistant with sound-localisation and speech-to-text application. International Journal of Medical Engineering and Informatics, 14(1), 63–73. 10.1504/ijmei.2022.119313

[ref24] Geers, A. E., Moog, J. S., Biedenstein, J., Brenner, C., & Hayes, H. (2009). Spoken language scores of children using cochlear implants compared to hearing age-mates at school entry. Journal of Deaf Studies and Deaf Education, 14(3), 371–385. 10.1093/deafed/enn046 19155289

[ref25] Geers, A. E., Nicholas, J., Tobey, E., & Davidson, L. (2016). Persistent language delay versus late language emergence in children with early cochlear implantation. Journal of Speech Language and Hearing Research, 59(1), 155–170. 10.1044/2015_JSLHR-H-14-0173 PMC4867929 26501740

[ref26] Girolametto, L. (1997). Development of a parent report measure for profiling the conversational skills of preschool children. American Journal of Speech-Language Pathology, 6(4), 25–33. 10.1044/1058-0360.0604.25

[ref28] Goberis, D., Beams, D., Dalpes, M., Abrisch, A., Baca, R., & Yoshinaga-Itano, C. (2012). The missing link in language development of deaf and hard of hearing children: Pragmatic language development. Seminars in Speech and Language, 33(4), 297–309. 10.1055/s-0032-1326916 23081790

[ref29] Goodman, R. (1997). The strengths and difficulties questionnaire: A research note. Journal of Child Psychology & Psychiatry, 38(5), 581–586. 10.1111/j.1469-7610.1997.tb01545.x 9255702

[ref30] Goodwin, C., & Heritage, J. (1990). Conversation analysis. Annual Review of Anthropology, 19, 283–307. 10.1146/annurev.an.19.100190.001435

[ref32] Hart, B., & Risley, T. R. (1995). In H. Paul (Ed.), Meaningful differences in the everyday experience of young American children. Brookes Publishing.

[ref33] Heine, C., & Browning, C. J. (2004). The communication and psychosocial perceptions of older adults with sensory loss: A qualitative study. Ageing and Society, 24(1), 113–130. 10.1017/s0144686x03001491

[ref34] Hilviu, D., Parola, A., Vivaldo, S., Di Lisi, D., Consolino, P., & Bosco, F. M. (2021). Children with hearing impairment and early cochlear implant: A pragmatic assessment. Heliyon, 7, e07428. 10.1016/j.heliyon.2021.e07428 34286120 PMC8273221

[ref36] Holzinger, D., & Fellinger, J. (2022). Conversation difficulties rather than language deficits are linked to emotional problems in school children with hearing loss. Frontiers in Education, 7. 10.3389/feduc.2022.944814

[ref40] Joanna Briggs Institute (2013). JBI levels of evidence. https://jbi.global/sites/default/files/2019-05/JBI-Levels-of-evidence_2014_0.pdf

[ref41] Joint Committee on Infant Hearing (2019). Year 2019 position statement: Principles and guidelines for early hearing detection and intervention programs. The Journal of Early Hearing Detection and Intervention, 4(2), 1–44. 10.15142/fptk-b748 10943021

[ref42] Kazemi, Y. (2007). Children's communication checklist: The study of Persian children. Journal of Research in Rehabilitation Sciences, 2, 1–5.

[ref44] Khodeir, M. S., Hegazi, M. A., & Saleh, M. M. (2017). Development and standardization of a test for pragmatic language skills in Egyptian Arabic: The Egyptian Arabic Pragmatic Language Test (EAPLT). Folia Phoniatrica et Logopedica, 69(5–6), 209–218. 10.1159/000485656 29554657

[ref48] Levac, D., Colquhoun, H., & O'Brien, K. K. (2010). Scoping studies: Advancing the methodology. Implement Science, 5(1), 69. 10.1186/1748-5908-5-69 PMC2954944 20854677

[ref49] Li, T., Higgins, J., & Deeks, J. (2022). Collecting Data. In J. Higgins, J. Thomas, J. Chandler, M. Cumpston, T. Li, M. Page & V. Welch (Eds.), Cochrane Handbook for Systematic Reviews of Interventions. Cochrane. https://training.cochrane.org/handbook/current/chapter-05.

[ref50] Lindsay, J., & Wilkinson, R. (1999). Repair sequences in aphasic talk: A comparison of aphasic-speech and language therapist and aphasic-spouse conversations. Aphasiology, 13(4–5), 305–325. 10.1080/026870399402118

[ref51] Lloyd, J., Lieven, E., & Arnold, P. (2001). Oral conversations between hearing-impaired children and their normally hearing peers and teachers. First Language, 21, 83–107. 10.1177/014272370102106104

[ref52] Lorusso, M. (2009). APL Medea: Abilita Pragmatiche Nel Linguaggio Medea. Firenze: Giunti OS.

[ref53] MacWhinney, B. (2000). The CHILDES project: Tools for analyzing talk: Transcription format and programs (3rd Ed.). Lawrence Erlbaum Associates.

[ref55] Matthews, D., & Kelly, C. (2022). Pragmatic development in deaf and hard of hearing children: A review. Deafness & Education International, 24(4), 296–313. 10.1080/14643154.2022.2140251

[ref56] McCreery, R. W., Walker, E. A., & Spratford, M. (2015). Understanding limited use of amplification in infants and children who are hard of hearing. Perspectives on Hearing and Hearing Disorders in Childhood, 25(1), 15–23. 10.1044/hhdc25.1.15

[ref57] Moeller, M. P., Hoover, B., Peterson, B., & Stelmachowicz, P. (2009). Consistency of hearing aid use in infants with early-identified hearing loss. American Journal of Audiology, 18(1), 14–23. 10.1044/1059-0889(2008/08-0010) 19029531 PMC2692469

[ref58] Moher, D., Shamseer, L., Clarke, M., Ghersi, D., Liberati, A., Petticrew, M., Shekelle, P., Stewart, L. A., & Group, P. P (2015). Preferred reporting items for systematic review and meta-analysis protocols (PRISMA-P) 2015 statement. Systematic Reviews, 4(1), 1. 10.1186/2046-4053-4-1 25554246 PMC4320440

[ref60] Most, T., Shina-August, E., & Meilijson, S. (2010). Pragmatic abilities of children with hearing loss using Cochlear implants or hearing aids compared to hearing children. Journal of Deaf Studies and Deaf Education, 15(4), 422–437. 10.1093/deafed/enq032 20624757

[ref61] Munn, Z., Peters, M. D. J., Stern, C., Tufanaru, C., McArthur, A., & Aromataris, E. (2018). Systematic review or scoping review? Guidance for authors when choosing between a systematic or scoping review approach. BMC Medical Research Methodology, 18(143), 143. 10.1186/s12874-018-0611-x 30453902 PMC6245623

[ref62] Nailand, L., Munro, N., & Purcell, A. (2021). Identifying the factors that affect consistent hearing aid use in young children early identified hearing loss: A scoping review. Ear and Hearing. 10.1097/aud.0000000000001139 34643596

[ref64] Paatsch, L. E., & Toe, D. M. (2014). A comparison of pragmatic abilities of children who are deaf or hard of hearing and their hearing peers. Journal of Deaf Studies and Deaf Education, 19(1), 1–19. 10.1093/deafed/ent030 23813695

[ref65] Peters, M. D., Godfrey, C. M., Khalil, H., McInerney, P., Parker, D., & Soares, C. B. (2015). Guidance for conducting systematic scoping reviews. International Journal of Evidence Based Healthcare, 13(3), 141–146. 10.1097/XEB.0000000000000050 26134548

[ref68] Petrisor, B., & Bhandari, M. (2007). The hierarchy of evidence: Levels and grades of recommendation. Indian Journal of Orthopaedics, 41(1), 11–15. https://doi.org.10.4103/0019-5413.30519, https://doi.org.10.4103/0019-5413.30519 21124676 10.4103/0019-5413.30519 PMC2981887

[ref69] Pollock, D., Peters, M. D. J., Khalil, H., McInerney, P., Alexander, L., Tricco, A. C., Evans, C., de Moraes, É. B., Godfrey, C. M., Pieper, D., Saran, A., Stern, C., & Munn, Z. (2023). Recommendations for the extraction, analysis, and presentation of results in scoping reviews. JBI Evidence Synthesis, 21(3), 520–532. 10.11124/JBIES-22-00123 36081365

[ref70] Prutting, C. A., & Kirchner, D. M. (1987). A clinical appraisal of the pragmatic aspects of language. Journal of Speech & Hearing Disorders, 52, 105–119. 10.1044/jshd.5202.105 3573742

[ref71] Remine, M. D., Brown, P. M., & Cowan, R. S. (2003). Assessing children with profound hearing loss and severe language delay: Getting a broader picture. Cochlear Implants International, 4(2), 73–84. 10.1179/cim.2003.4.2.73 18792139

[ref73] Rinaldi, P., Baruffaldi, F., Burdo, S., & Caselli, M. C. (2013). Linguistic and pragmatic skills in toddlers with cochlear implant. International Journal of Language & Communication Disorders, 48(6), 715–725. 10.1111/1460-6984.12046 24165367

[ref74] Sacks, H., Schegloff, E. A., & Jefferson, G. (1974). A simplest systematics for the organization of turn-taking for conversation. Language, 50(4), 696–735. 10.1353/lan.1974.0010

[ref77] Schegloff, E. A. (1992). Repair after next turn: The last structurally provided defense of intersubjectivity in conversation. The American Journal of Sociology, 97(5), 1295–1345. 10.1086/229903

[ref78] Semel, E. M., Wiig, E. H., & Secord, W. (2003). Clinical Evaluation of Language Fundamentals (4th Ed.). Psychological Corp.

[ref79] Shoeib, R. M., Kaddah, F. E.-Z. A., Kheir El-Din, S. T., & Said, N. M. (2016). Study of pragmatic language ability in children with hearing loss. The Egyptian Journal of Otolaryngology, 32(3), 210–218. 10.4103/1012-5574.186526

[ref80] Socher, M., Lyxell, B., Ellis, R., Garskog, M., Hedstrom, I., & Wass, M. (2019). Pragmatic language skills: A comparison of children with cochlear implants and children without hearing loss. Frontiers in Psychology, 10, 2243. 10.3389/fpsyg.2019.02243 31649586 PMC6794448

[ref81] Stephens, S. D., Jaworski, A., Lewis, P., & Aslan, S. (1999). An analysis of the communication tactics used by hearing-impaired adults. British Journal of Audiology, 33(1), 17–27. 10.3109/03005364000000097 10219720

[ref83] The Joanna Briggs Institute (2017). Checklist for analytical cross sectional studies. https://jbi.global/sites/default/files/2019-05/JBI_Critical_Appraisal-Checklist_for_Analytical_Cross_Sectional_Studies2017_0.pdf

[ref84] Tobey, E. A., Thal, D., Niparko, J. K., Eisenberg, L. S., Quittner, A. L., Wang, N. Y., & Team, C. D. I (2013). Influence of implantation age on school-age language performance in pediatric cochlear implant users. International Journal of Audiology, 52(4), 219–229. 10.3109/14992027.2012.759666 23448124 PMC3742378

[ref85] Toe, D., Beattie, R., & Barr, M. (2007). The development of pragmatic skills in children who are severely and profoundly deaf. Deafness & Education International, 9(2), 101–117. 10.1179/146431507790560011

[ref87] Toe, D. M., & Paatsch, L. E. (2013). The conversational skills of school-aged children with cochlear implants. Cochlear Implants International, 14(2), 67–79. 10.1179/1754762812Y.0000000002 23453220

[ref90] Tomblin, J. B., Oleson, J., Ambrose, S. E., Walker, E. A., McCreery, R. W., & Moeller, M. P. (2020). Aided hearing moderates the academic outcomes of children with mild to severe hearing loss. Ear and Hearing, 41(4), 775–789. 10.1097/AUD.0000000000000823 32032223 PMC7546580

[ref91] Tomblin, J. B., Oleson, J. J., Ambrose, S. E., Walker, E., & Moeller, M. P. (2014). The influence of hearing aids on the speech and language development of children with hearing loss. JAMA Otolaryngology–Head & Neck Surgery, 140(5), 403–409. 10.1001/jamaoto.2014.267 24700303 PMC4066968

[ref92] Tricco, A. C., Lillie, E., Zarin, W., O'Brien, K. K., Colquhoun, H., Levac, D., Moher, D., Peters, M. D. J., Horsley, T., Weeks, L., Hempel, S., Akl, E. A., Chang, C., McGowan, J., Stewart, L., Hartling, L., Aldcroft, A., Wilson, M. G., Garritty, C. et al. (2018). PRISMA extension for scoping reviews (PRISMA-ScR): Checklist and explanation. Annals of Internal Medicine, 169(7), 467–473. 10.7326/M18-0850 30178033

[ref93] Tye-Murray, N. (2003). Conversational fluency of children who use cochlear implants. Ear and Hearing, 24(1), 82S–89S. 10.1097/01.AUD.0000051691.33869.EC 12612483

[ref94] Walker, E. A., Spratford, M., Moeller, M. P., Oleson, J., Ou, H., Roush, P., & Jacobs, S. (2013). Predictors of hearing aid use time in children with mild-to-severe hearing loss. Language, Speech & Hearing Services in Schools, 44(1), 73–88. 10.1044/0161-1461(2012/12-0005) 22869089 PMC3543484

[ref95] World Health Organization (2024). Deafness and hearing loss. [Fact Sheet]. https://www.who.int/news-room/fact-sheets/detail/deafness-and-hearing-loss#:~:text='Hard%20of%20hearing'%20refers%20to,devices%20as%20well%20as%20captioning

[ref96] Xie, Y.-H., Potmesil, M., & Farrow, B. (2014). Children who are deaf or hard of hearing in inclusive educational settings: A literature review on interactions with peers. Journal of Deaf Studies and Deaf Education, 19, 423–437. 10.1093/deafed/enu017 25052819

[ref97] Yoshinaga-Itano, C., Baca, R. L., & Sedey, A. L. (2010). Describing the trajectory of language development in the presence of severe-to-profound hearing loss: A closer look at children with cochlear implants versus hearing aids. Otology & Neurotology, 31(8), 1268–1274. 10.1097/MAO.0b013e3181f1ce07 20818291 PMC3014847

[ref99] Yoshinaga-Itano, C., Sedey, A. L., Wiggin, M., & Chung, W. (2017). Early hearing detection and vocabulary of children with hearing loss. Pediatrics, 140(2), e20162964. 10.1542/peds.2016-2964 PMC5595069 28689189

[ref100] Yoshinaga-Itano, C., & Wiggin, M. (2016). A look into the crystal ball for children who are deaf or hard of hearing: Needs, opportunities and challenges. Seminars in Speech and Language, 37(4), 252–258. 10.1055/s-0036-1587707 27701701

[ref101] Zaidman-Zait, A., & Most, T. (2020). Pragmatics and peer relationships among deaf, hard of hearing, and hearing adolescents. Pediatrics, 146(Suppl 3), S298–S303. 10.1542/peds.2020-0242J 33139444

