# Supplementary material for: Conversation and pragmatics in children who are hard-of-hearing: a scoping review
Source: J Deaf Stud Deaf Educ. 2024 May 16;29(4):456–66. doi: 10.1093/deafed/enae011 (PMC11413802; doi:10.1093/deafed/enae011)
Supplement: Appendix_1_enae011 [file appendix_1_enae011.docx]

**Appendix 1**

***Ovid MEDLINE Search Strategy***

| Set | Search Statement |
| --- | --- |
| 1. | adolescent/ or child/ or child, preschool/ or infant/ |
| 2. | child*.mp. |
| 3. | p?ediatric*.mp. |
| 4. | infant*.mp. |
| 5. | adolescent*.mp. |
| 6. | school age*.mp. |
| 7. | preschool*.mp. |
| 8. | preschool age*.mp. |
| 9. | 1 or 2 or 3 or 4 or 5 or 6 or 7 or 8 |
| 10. | communication/ or language/ or nonverbal communication/ or verbal behavior/ or speech/ |
| 11. | child development/ or language development/ |
| 12. | communicat*.mp. |
| 13. | (listen and speak).mp. [mp=title, abstract, original title, name of substance word, subject heading word, floating sub-heading word, keyword heading word, organism supplementary concept word, protocol supplementary concept word, rare disease supplementary concept word, unique identifier, synonyms] |
| 14. | talk*.mp. |
| 15. | speak*.mp. |
| 16. | oral communication.mp. |
| 17. | oral language.mp. |
| 18. | speech.mp. |
| 19. | spoken language.mp. |
| 20. | language.mp. |
| 21. | verbal.mp. |
| 22. | 10 or 11 or 12 or 13 or 14 or 15 or 16 or 17 or 18 or 19 or 20 or 21 |
| 23. | Hearing Aids/ or Cochlear Implants/ |
| 24. | hearing aid*.mp. |
| 25. | hearing device*.mp. |
| 26. | cochlear implant*.mp. |
| 27. | 23 or 24 or 25 or 26 |
| 28. | hearing loss*.mp. |
| 29. | hearing impair*.mp. |
| 30. | deaf.mp. |
| 31. | hard of hearing.mp. |
| 32. | congenital.mp. |
| 33. | pre$lingual.mp. |
| 34. | permanent.mp. |
| 35. | bilateral.mp. |
| 36. | from birth.mp. |
| 37. | born with.mp. |
| 38. | 28 or 29 or 30 or 31 or 32 or 33 or 34 or 35 or 36 or 37 |
| 39. | convers*.mp. |
| 40. | pragmatic*.mp. |
| 41. | social language.mp. |
| 42. | 39 or 40 or 41 |
| 43. | 9 and 22 and 27 and 38 and 42 |
| 44. | limit 43 to (English language and yr=”2000-2022”) |
