# Supplementary material for: Conversation and pragmatics in children who are hard-of-hearing: a scoping review
Source: J Deaf Stud Deaf Educ. 2024 May 16;29(4):456–66. doi: 10.1093/deafed/enae011 (PMC11413802; doi:10.1093/deafed/enae011)
Supplement: Appendix_2_enae011 [file appendix_2_enae011.docx]

**Appendix *2***

***JBI Quality Appraisal***

| Title & Author/s | Were the criteria for inclusion in the sample clearly defined? | Were the study subjects and the setting described in detail? | Was the exposure measured in a valid and reliable way? | Were objective, standard criteria used for measurement of the condition? | Were confounding factors identified? | Were strategies to deal with confounding factors stated? | Were the outcomes measured in a valid and reliable way? | Was appropriate statistical analysis used? |
| --- | --- | --- | --- | --- | --- | --- | --- | --- |
| Some trouble with repair: Conversations between children with cochlear implants and hearing peers  (Church et al., 2017) | No | No | No | Yes | No | No | No | No |
| A longitudinal study of pragmatic language development in three children with cochlear implants  (Dammeyer, 2013) | No | Yes | Yes | Yes | No | No | Yes | Yes |
| Peer interactions of preschool children with and without hearing loss  (DeLuzio & Girolametto, 2011) | No | Yes | Yes | Yes | Yes | Yes | Yes | Yes |
| What's that you say? Communication breakdowns and their repairs in children who are deaf or hard of hearing  (Fitzpatrick et al., 2020) | No | Yes | Yes | Yes | No | Yes | Yes | Yes |
| The missing link in language development of deaf and hard of hearing children: pragmatic language development  (Goberis et al., 2012) | No | No | Yes | Yes | No | No | No | No |
| Social conversational skills development in early implanted children  (Guerzoni et al., 2016) | No | Yes | No | Yes | Yes | Yes | Yes | Yes |
| Children with hearing impairment and early cochlear implant: A pragmatic assessment  (Hilviu et al., 2021) | No | Yes | Yes | Yes | Yes | Yes | Yes | Yes |
| Discourse strategies and the production of prosody by prelingually deaf adolescent cochlear implant users  (Holt et al., 2017) | No | Yes | Yes | Yes | No | No | Yes | Yes |
| Speech recognition working memory and conversation in children with cochlear implants  (Ibertsson et al., 2009a) | No | No | Yes | Yes | No | No | Yes | Yes |
| Deaf teenagers with cochlear implants in conversation with hearing peers  (Ibertsson et al., 2009b) | No | No | Yes | Yes | No | No | Yes | Yes |
| The pragmatic skills of profoundly deaf children  (Jeanes et al., 2000) | No | No | Yes | Yes | Yes | No | Yes | Yes |
| Early pragmatics in deaf and hard of hearing infants  (Kelly et al., 2020) | No | Yes | Yes | Yes | Yes | Yes | Yes | Yes |
| The effect of age at time of cochlear implantation on the pragmatic development of the prelingual hearing impaired children  (Khodeir et al., 2021) | No | No | No | Yes | No | No | Yes | Yes |
| Development of implanted deaf children's conversational skills  (Le Maner-Idrissi et al., 2010) | No | Yes | Yes | Yes | Yes | No | Yes | Yes |
| Conversations between deaf children and their hearing mothers: pragmatic and dialogic characteristics  (Lederberg & Everhart, 2000) | No | No | No | Yes | No | No | Yes | Yes |
| Oral conversations between hearing-impaired children and their normally hearing peers and teachers  (Lloyd et al., 2001) | No | No | Yes | Yes | No | No | Yes | Yes |
| Adequate formal language performance in unilateral cochlear implanted children: is it indicative of complete recovery in all linguistic domains? Insights from referential communication  (Mancini et al., 2015) | Yes | Yes | Yes | Yes | Yes | No | Yes | Yes |
| The use of repair strategies by children with and without hearing impairment  (Most, 2002) | No | Yes | Yes | Yes | No | No | Yes | Yes |
| Pragmatic abilities of children with hearing loss using cochlear implants or hearing AIDS compared to hearing children  (Most et al., 2010) | No | Yes | Yes | Yes | No | No | Yes | Yes |
| Hearing status language modality and young children's communicative and linguistic behavior  (Nicholas & Geers, 2003) | No | Yes | Yes | Yes | No | No | Yes | Yes |
| A comparison of pragmatic abilities of children who are deaf or hard of hearing and their hearing peers  (Paatsch & Toe, 2014) | No | Yes | No | Yes | Yes | No | Yes | Yes |
| Assessing children with profound hearing loss and severe language delay: getting a broader picture  (Remine et al., 2003) | No | Yes | No | Yes | Yes | No | No | N/A |
| Pragmatic skills in children with hearing loss: comparison between cochlear implants and hearing aids users  (Rezaei et al., 2021) | No | No | No | Yes | No | No | No | Yes |
| Linguistic and pragmatic skills in toddlers with cochlear implant  (Rinaldi et al., 2013) | No | Yes | Yes | Yes | Yes | Yes | Yes | Yes |
| Clarification requests in everyday interaction involving children with cochlear implants  (Samuelsson & Lyxell, 2014) | No | No | No | Yes | No | No | No | Yes |
| You sometimes get more than you ask for': responses in referential communication between children and adolescents with cochlear implant and hearing peers  (Sandgren et al., 2011) | No | No | Yes | Yes | No | No | Yes | Yes |
| Study of pragmatic language ability in children with hearing loss  (Shoeib et al., 2016) | Yes | Yes | Yes | Yes | No | No | Yes | Yes |
| Pragmatic language skills: A comparison of children with cochlear implants and children without hearing loss  (Socher et al., 2019) | No | Yes | Yes | Yes | No | No | No | Yes |
| Pragmatic language in deaf and hard of hearing students: correlation with success in general education  (Thagard et al., 2011) | No | No | Yes | Yes | Yes | No | Yes | Yes |
| Communicative competence of oral deaf children while explaining game rules  (Toe & Paatsch, 2018) | No | No | Yes | Yes | No | No | Yes | Yes |
| The conversational skills of school-aged children with cochlear implants  (Toe & Paatsch, 2013) | No | Yes | Yes | Yes | No | No | Yes | Yes |
| The communication skills used by deaf children and their hearing peers in a question-and-answer game context  (Toe & Paatsch, 2010) | No | Yes | Yes | Yes | No | No | Yes | Yes |
| The development of pragmatic skills in children who are severely and profoundly deaf  (Toe et al., 2013) | No | Yes | Yes | Yes | No | No | Yes | Yes |
| Conversational fluency of children who use cochlear implants  (Tye-Murray, 2003) | No | No | No | Yes | Yes | Yes | Yes | Yes |
| Early intervention parent talk and pragmatic language in children with hearing loss  (Yoshinaga-Itano et al., 2020) | No | No | No | Yes | No | No | No | Yes |
| Pragmatics and peer relationships among deaf hard of hearing and hearing adolescents  (Zaidman-Zait & Most, 2020) | No | Yes | No | Yes | No | Yes | No | Yes |
