# Supplementary material for: Conversation and pragmatics in children who are hard-of-hearing: a scoping review
Source: J Deaf Stud Deaf Educ. 2024 May 16;29(4):456–66. doi: 10.1093/deafed/enae011 (PMC11413802; doi:10.1093/deafed/enae011)
Supplement: Appendix_4_enae011 [file appendix_4_enae011.docx]

**Appendix 4**

***Data Extraction Instrument: Studies about Pragmatics***

| Title & Author/s | Country / Language | Objectives | Participants | Context | Outcomes |
| --- | --- | --- | --- | --- | --- |
| A longitudinal study of pragmatic language development in three children with cochlear implants  (Dammeyer, 2013) | Australia (English) | To observe speech intelligibility, auditory performance, turn taking, repair behaviors and gaze (aspects of pragmatics) in HoH children over time. | 3 HoH children with CIs. Average age 60 months (range 57-63 months) at first recording. Average IA = 24 months (range 18–29 months). | Children observed in natural interactions (90 mins of free conversation) 6 times / year for 3 years, then 2 times in the final year (total duration of 4 years). Self-developed schemas were used to assess skills in the observed conversations. | Children improved their speech intelligibility and auditory performance during the 4 years, but they continued to have difficulties with pragmatic language usage. |
| Peer interactions of preschool children with and without hearing loss  (DeLuzio & Girolametto, 2011) | Canada (English) | To understand how children with severe to profound HL develop social interaction skills by investigating how they manage conversational exchanges with TH peers. | 12 HoH children CA range 37-62 months (average CA 49.3 month) with severe to profound HL, 6 using CIs and 6 used bilateral HAs, compared with 12 TH children matched for intelligence, language, speech, and social skill development.  Diagnosis made before 24 months of age. | Observations of Initiation and response skills during 20 minutes of group play. Integrated preschool programs.  Outcome measures included number and type of initiation strategies, number of responses, and length of interactions. Self-developed schema used. | HoH children had poorer speech, language, and social development than TH peers overall, however, there were no significant differences in initiation and response skills between groups.  Having age-appropriate language skills did not ensure successful peer interactions. HoH preschool children were excluded from interactions by their playmates.  Playmates initiated interactions with HoH children less often than with other TH children. TH children ignored initiations from HoH children more often than those of other TH children. |
| The missing link in language development of deaf and hard of hearing children: pragmatic language development  (Goberis et al., 2012) | America (English) | To describe pragmatic development of TH and HoH children. | 126 HoH children from 2-7 years, with CIs, compared with 109 TH age-matched peers from 2-7 years.  CA range from 26–69 months, average IA 41 months (range = 26–69).  Data analyzed by age groups,  18 to 29 months, 30 to 41 months, 42 to 53 months, 54 to 65 months, 66 to 77 months, 78 to 89 months, and 90 months.  Between 30 and 50 participants (HoH and TH) included in each age grouping. | All children had normal cognitive development.  Degree of HL varied; 19.8% of HoH children had mild HL, 24.2% had moderate to moderate-severe HL, 32.9% had severe HL, and 23.1% had profound HL.  Skills measured using 'The Pragmatics Checklist'. (Goberis, 1999). | TH children mastered 44% (20 of 45) of items using ‘complex language’ by CA3, 95.5% (43 of 45) of items by CA, 98% of items by CA5, and 100% of items by CA6.  HoH children mastered 6.6% (3 of 45) of the items with ‘complex language’ by CA6, and 69% (31 of 45) of items by CA7.  HoH children were significantly older than TH peers when they demonstrated items on the assessment with ‘complex language’. |
| Children with hearing impairment and early cochlear implant: A pragmatic assessment  (Hilviu et al., 2021) | Italy (Italian) | To understand the relationship between age at CI, and development of pragmatic skills in HoH children. | 18 HoH children who received CI before CA2, compared with TH peers matched by age. All otherwise typically developing.  Assessments completed for three groups aged 6;11 to 7;11, 8;0 to 8;11, and 9;0 to 9;11 years. | Measured areas of pragmatics including understanding metaphors, implicit meaning, comics, situations using ‘The Pragmatic Language Skills Test’ (APL-Medea) (Lorusso, 2009) which includes measuring perspective taking using ‘The Colors Game’. | HoH children with CI achieved lower scores than TH peers.  HoH children with CIs differed from TH kids in comics, and colors game tasks.  Age at implantation was a moderate but significant predictor of pragmatic performance. |
| Early pragmatics in deaf and hard of hearing infants  (Kelly et al., 2020) | UK (English) | To understand whether HoH infants are more or less likely to engage in gestural and vocal pragmatic behaviors, compared with normative info on 5 types of infant commination known to positively predict later language development (show gestures, give gestures, index finger pointing, communicative vocalizations and early word use). | 8 HoH infants aged 12-18 months old, with  HL ranging from moderate to profound HL. Infants received HAs at between 5 and 15 weeks old. No additional needs, not premature or low birth weight.  Most families used spoken language only (but mixture of spoken and sign-supported).  More male than females in sample.  Compared with 8 TH infants matched for age, gender, and SES. | Observing natural interactions, free play with 25 mins at home was recorded, and then self-developed checklist was administered.  Analyzed the frequency of use of 5 types of infant communication known to positively predict later language development (i.e., show gestures, give gestures, index-finger pointing, communicative vocalizations, and early word use). | Hearing loss had a significant negative effect on how frequently infants engaged in all types of early communication that can predict later language development.  HoH infants are at high risk of delay in the gestural and vocal communicative skills that lay the foundations for later language. Delays both in gestural and vocal domains suggests that it’s not just HL, but interactive experiences that are impacted. |
| The effect of age at time of cochlear implantation on the pragmatic development of the prelingual hearing impaired children  (Khodeir et al., 2021) | Egypt (Arabic) | To understand whether age at CI (pre/post 3 years old) influences pragmatic language development in HoH children. | 60 HoH Arabic speaking children between 4 and 7;4 years, with pre lingual bilateral severe to profound SNHL and ‘normal auditory levels’ by CI aided audiogram.  All children had 6 months post-CI rehab.  30 children implanted before 3 years and 30 implanted after 3 years old. | Egyptian Arabic Pragmatic Language Test (EAPLT) (Khodeir et al., 2017). Scores at 5^th^ %ile interpreted as ‘child has acquired the skill’, while scores at 95^th^ %ile interpreted as 'child has mastered the skill'.  Average CA at diagnosis for PRE 3 years group was 8 months (average age when HAs fitted was 2.3 years), and POST 3 years group was 1.9 years (average when HAs fitted was 4.7 years).  Compared CI before 3 years old, with CI after 3 years old. | HoH children had poor pragmatic skills whether they received CI before 3 years or after 3 years.  Scores of the EAPLT were positively correlated to child’s CA, their language abilities, and duration of language rehabilitation provided. No significant correlation between EAPLT and CA at implantation. |
| Adequate formal language performance in unilateral cochlear implanted children: is it indicative of complete recovery in all linguistic domains? Insights from referential communication  (Mancini et al., 2015) | Italy (Italian) | To understand referential communication skills in HoH children with severe/profound HL. | 31 HoH children with unilateral CIs (in presence of bilateral loss, some had contralateral HA), and language development WNL.  Age at diagnoses ranged from 4 months to 36 months, various etiology. Age at CI ranged from 9 months to 41 months, and IA ranged from 69 months to 127 months. | Pragmatics skills assessed with ‘The Pragmatic Language Skills Test’ (APL-Medea) (Lorusso, 2009) including ‘The Colors Game’ played with their parent.  Game scored on a 3-poin scale, in five areas; description of materials, step rule, joker function, black face function, and how to win. Normative data available. | 83.9% HoH children performed appropriately for their CA.  CI had a positive effect on referential communication, although some CI users continued struggle. |
| Pragmatic abilities of children with hearing loss using cochlear implants or hearing AIDS compared to hearing children  (Most et al., 2010) | Israel (Hebrew) | To characterize pragmatic abilities of HoH children (using HAs or CIs). | 24 HoH children, 13 using HAs, and 11 using CIs. Average CA = 91 months (range = 79–103 months), average IA = 30 months (range = 14–60 months.  Compared with 13 TH children average CA = 88 months (range 68-112 months), matched on language age. | 15-min free sample used to inform completion of The Pragmatic Protocol (Prutting & Kirchner, 1987).  HoH children used spoken language, attended mainstream schools, and accessed communication therapy twice a week.  Results from HoH children using CIs and HAs, compared to results from TH peers. | More inappropriate pragmatic behaviors were used by > 50% of HoH children (n = 10) compared with TH children (n = 2). |
| Pragmatic skills in children with hearing loss: comparison between cochlear implants and hearing aids users  (Rezaei et al., 2021) | Iran (Persian) | To understand whether HoH children with CIs have better pragmatic skills than HoH children with HAs. | 52 children in 3 groups, HoH CI users (n = 16), HoH HA users (n = 16), TH children (n = 20).  Average CA was 6 years old. | Persian version of the Children’s Communication Checklist (Kazemi, 2007). | HoH children had acceptable pragmatic skills compared with TH peers based on parent-report.  Suggests that HoH children at early stages of language development can develop pragmatic skills despite language delay. |
| Linguistic and pragmatic skills in toddlers with cochlear implant  (Rinaldi et al., 2013) | Italy (Italian) | To compare linguistic skills of children implanted by 12 months of age with children implanted between 13 and 26 months of age; and to describe the relationship among lexical, grammar and pragmatic skills. | 12 HoH children with CIs, average CA 29 months (range = 24–34 months), average IA = 15 (range = 9–26).  Compared with normative data | Assessment - Le Abilità Socio- Conversazionali del Bambino (Girolametto, 1997). | Most children with CIs performed below the mean, with many outside the normal range. CI may provide HoH children with opportunity to develop language skills, but difficulties in early social experiences and interaction remains. |
| Study of pragmatic language ability in children with hearing loss  (Shoeib et al., 2016) | Egypt (Arabic) | To examine the pragmatic abilities of HoH children with SNHL with goal to develop intervention program to mitigate effect of early pragmatic difficulties on later academic and social abilities. | 27 HoH children, HL severities including mild (n = 9), moderate (n = 8), moderately severe (n = 5), and severe (n = 5) loss. Average PTA 61.04 (+/1 15.14 dB). Average CA 8.78 years (+/- 2.04 years, range 30-80dB).  All children using bilateral HAs with 'satisfactorily aided responses’. All used spoken language ‘sufficiently well’ to qualify for the study.  Compared with 27 TH age and gender matched peers. | Used Arabic versions of Test of Pragmatic Language, Observational Rating Scale and Pragmatic Profile subsets of the CELF-4 (Alduais, 2012). Also collected standardized language scores.  Children had no other disabilities and did not use sign language. 16 children were in regular classes. | Significantly lower pragmatic abilities in children with HL compared with children with TH.  Male HoH children worse than female HoH children.  Significant correlations between pragmatic variables and degree of HL, speech discrimination ability, and the duration of auditory deprivation. |
| Pragmatic language skills: A comparison of children with cochlear implants and children without hearing loss  (Socher et al., 2019) | Sweden (Swedish) | To compare pragmatic skills of HoH children with CIs to those with TH hearing. | 14 HoH children with CIs from age 5;7 to 8;11 years (preschool, first and second graders). 10 girls, 4 boys, average CA6.77 years (SD = 11.13 months).  Diagnosis made at average age of 11.14 months (SD = 13.84 months), 3 children unilateral CI, 11 bilateral CI. Average age at implantation 24.07 months (SD = 19.55 months).  2 children bilingual (sign language and oral language), 4 children used only oral language, 8 used oral language mainly and signs for support.  Compared with 34 TH children matched by age. | HoH children from special school and hearing clinic in Sweden, TH children from local mainstream school.  The Pragmatics Profile of the CELF-4 (Semel et al., 2003) to assess pragmatics, other assessments used to measure vocabulary, grammar, and other verbal cognitive measures. | Many HoH children with CI show pragmatic language ability like their TH peers.  Significant differences were found on a pragmatic measure connected to theory of mind which has been found to be delayed in deaf and hard of hearing children.  Verbal fluency correlated with all three sub-measures of pragmatic language ability in the Pragmatics Profile (causal direction is unclear). |
| Pragmatic language in deaf and hard of hearing students: correlation with success in general education  (Thagard et al., 2011) | America (English) | To understand the relationship between socio-linguistic pragmatic competence in HoH students, and the student’s degree of loss, communication mode, and their degree of success in general education using self-developed checklists. | 81 HoH children from preschool to grade 8, mixture of spoken and signed language users, range of degree of HL, 1/3 used CIs, 2/3 used HAs. 59 used spoken language and the remaining 22 used signed language.  No comparison group. | Self-developed measures used. | Better socio-pragmatic language skills were highly positively correlated with academic outcomes in HoH children, regardless of communication modality (spoken or signed language). |
| Early intervention parent talk and pragmatic language in children with hearing loss  (Yoshinaga-Itano et al., 2020) | America (English) | To identify variables associated with pragmatic language ability in HoH children. | 124 HoH children with bilateral HL from 4-7 years old, compared to normative data previously reported on ‘mastery’ of skills by TH children.  All used spoken English, 16% also used sign language with spoken language. Most had no additional disabilities, 10% did report additional disabilities. Range of non-verbal IQs, 4% scored >2SDs below average.  Degree of HL ranged from mild to profound, all used HAs or CIs. 51% were male, 84% primarily spoken language. | Pragmatic skills evaluated annually using ‘The Pragmatics Checklist’ (Goberis, 1999).  Cohort of children in Colorado. | HoH children who met 1-3-6 guidelines (Joint Committee on Infant Hearing, 2019) scored higher on this measure than HoH children who did not meet 1-3-6 guidelines (Joint Committee on Infant Hearing, 2019).  HoH children who were a) exposed to more parent talk, b) had higher non-verbal intelligence, c) lesser degree of HL and d) whose mothers were more educated, also did better. |
| Pragmatics and peer relationships among deaf hard of hearing and hearing adolescents  (Zaidman-Zait & Most, 2020) | Israel (Hebrew) | 1. To understand differences in pragmatic abilities and peer relationships between HoH adolescents and TH peers. 2. To explore the contribution of pragmatic skills and speech (i.e., articulation and intelligibility) to social aspects of school functioning. | 33 HoH adolescents with moderate to profound HL from grades 7-11 (57% male, average CA 14.83 years SD 1.12), compared with 34 TH adolescents (53% boys, average CA 14.86 years, SD 1.18) also from grades 7-11.  62% used HAs, and 38% CIs. Overall, 86.7 were bilaterally aided (remainder unilaterally aided). | Used teacher reports on ‘Strengths and Difficulties Questionnaire’ (SDQ) (Goodman, 1997) to look at prosocial behaviors and peer relationships, and The Children’s Communication Checklist-2 (CCC-2) (Bishop, 2003) to assess pragmatic language. Also gathered self-reported info on supportiveness of peer relationship and school engagement. | HoH adolescents had more difficulty interacting with peers than did their TH peers. HoH adolescents demonstrated more pragmatic and speech difficulties than hearing peers, and higher levels of peer relationship problems and less prosocial behavior that their TH peers.  Positive correlation between pragmatic abilities and success in peer relationships and more prosocial behaviors for both TH and HoH participants.  Better pragmatic skills positively correlated with adolescents’ perceptions of peer support, and better speech intelligibility associated with higher levels of school engagement. |

**Abbreviations:** HA = hearing aid/s, HL = hearing loss, CI = cochlear implant/s, CA = chronological age, HoH = deaf and hard-of-hearing, TH = typical/ly hearing, SD = standard deviation, IA = implant age, TODs = teachers of the deaf, SNHL = sensorineural hearing loss, SN = sensorineural, PTA = pure tone average, SES = socio-economic status, RI = repair initiator / initiations
