# Supplementary material for: Conversation and pragmatics in children who are hard-of-hearing: a scoping review
Source: J Deaf Stud Deaf Educ. 2024 May 16;29(4):456–66. doi: 10.1093/deafed/enae011 (PMC11413802; doi:10.1093/deafed/enae011)
Supplement: All_appendices-Revised_enae011 [file all_appendices-revised_enae011.docx]

**Appendix 1**

*JBI Quality Appraisal*

| Title & Author/s | Were the criteria for inclusion in the sample clearly defined? | Were the study subjects and the setting described in detail? | Was the exposure measured in a valid and reliable way? | Were objective, standard criteria used for measurement of the condition? | Were confounding factors identified? | Were strategies to deal with confounding factors stated? | Were the outcomes measured in a valid and reliable way? | Was appropriate statistical analysis used? |
| --- | --- | --- | --- | --- | --- | --- | --- | --- |
| Some trouble with repair: Conversations between children with cochlear implants and hearing peers  (Church et al., 2017) | No | No | No | Yes | No | No | No | No |
| A longitudinal study of pragmatic language development in three children with cochlear implants  (Dammeyer, 2013) | No | Yes | Yes | Yes | No | No | Yes | Yes |
| Peer interactions of preschool children with and without hearing loss  (DeLuzio & Girolametto, 2011) | No | Yes | Yes | Yes | Yes | Yes | Yes | Yes |
| What's that you say? Communication breakdowns and their repairs in children who are deaf or hard of hearing  (Fitzpatrick et al., 2020) | No | Yes | Yes | Yes | No | Yes | Yes | Yes |
| The missing link in language development of deaf and hard of hearing children: pragmatic language development  (Goberis et al., 2012) | No | No | Yes | Yes | No | No | No | No |
| Social conversational skills development in early implanted children  (Guerzoni et al., 2016) | No | Yes | No | Yes | Yes | Yes | Yes | Yes |
| Children with hearing impairment and early cochlear implant: A pragmatic assessment  (Hilviu et al., 2021) | No | Yes | Yes | Yes | Yes | Yes | Yes | Yes |
| Discourse strategies and the production of prosody by prelingually deaf adolescent cochlear implant users  (Holt et al., 2017) | No | Yes | Yes | Yes | No | No | Yes | Yes |
| Speech recognition working memory and conversation in children with cochlear implants  (Ibertsson et al., 2009a) | No | No | Yes | Yes | No | No | Yes | Yes |
| Deaf teenagers with cochlear implants in conversation with hearing peers  (Ibertsson et al., 2009b) | No | No | Yes | Yes | No | No | Yes | Yes |
| The pragmatic skills of profoundly deaf children  (Jeanes et al., 2000) | No | No | Yes | Yes | Yes | No | Yes | Yes |
| Early pragmatics in deaf and hard of hearing infants  (Kelly et al., 2020) | No | Yes | Yes | Yes | Yes | Yes | Yes | Yes |
| The effect of age at time of cochlear implantation on the pragmatic development of the prelingual hearing impaired children  (Khodeir et al., 2021) | No | No | No | Yes | No | No | Yes | Yes |
| Development of implanted deaf children's conversational skills  (Le Maner-Idrissi et al., 2010) | No | Yes | Yes | Yes | Yes | No | Yes | Yes |
| Conversations between deaf children and their hearing mothers: pragmatic and dialogic characteristics  (Lederberg & Everhart, 2000) | No | No | No | Yes | No | No | Yes | Yes |
| Oral conversations between hearing-impaired children and their normally hearing peers and teachers  (Lloyd et al., 2001) | No | No | Yes | Yes | No | No | Yes | Yes |
| Adequate formal language performance in unilateral cochlear implanted children: is it indicative of complete recovery in all linguistic domains? Insights from referential communication  (Mancini et al., 2015) | Yes | Yes | Yes | Yes | Yes | No | Yes | Yes |
| The use of repair strategies by children with and without hearing impairment  (Most, 2002) | No | Yes | Yes | Yes | No | No | Yes | Yes |
| Pragmatic abilities of children with hearing loss using cochlear implants or hearing AIDS compared to hearing children  (Most et al., 2010) | No | Yes | Yes | Yes | No | No | Yes | Yes |
| Hearing status language modality and young children's communicative and linguistic behavior  (Nicholas & Geers, 2003) | No | Yes | Yes | Yes | No | No | Yes | Yes |
| A comparison of pragmatic abilities of children who are deaf or hard of hearing and their hearing peers  (Paatsch & Toe, 2014) | No | Yes | No | Yes | Yes | No | Yes | Yes |
| Assessing children with profound hearing loss and severe language delay: getting a broader picture  (Remine et al., 2003) | No | Yes | No | Yes | Yes | No | No | N/A |
| Pragmatic skills in children with hearing loss: comparison between cochlear implants and hearing aids users  (Rezaei et al., 2021) | No | No | No | Yes | No | No | No | Yes |
| Linguistic and pragmatic skills in toddlers with cochlear implant  (Rinaldi et al., 2013) | No | Yes | Yes | Yes | Yes | Yes | Yes | Yes |
| Clarification requests in everyday interaction involving children with cochlear implants  (Samuelsson & Lyxell, 2014) | No | No | No | Yes | No | No | No | Yes |
| You sometimes get more than you ask for': responses in referential communication between children and adolescents with cochlear implant and hearing peers  (Sandgren et al., 2011) | No | No | Yes | Yes | No | No | Yes | Yes |
| Study of pragmatic language ability in children with hearing loss  (Shoeib et al., 2016) | Yes | Yes | Yes | Yes | No | No | Yes | Yes |
| Pragmatic language skills: A comparison of children with cochlear implants and children without hearing loss  (Socher et al., 2019) | No | Yes | Yes | Yes | No | No | No | Yes |
| Pragmatic language in deaf and hard of hearing students: correlation with success in general education  (Thagard et al., 2011) | No | No | Yes | Yes | Yes | No | Yes | Yes |
| Communicative competence of oral deaf children while explaining game rules  (Toe & Paatsch, 2018) | No | No | Yes | Yes | No | No | Yes | Yes |
| The conversational skills of school-aged children with cochlear implants  (Toe & Paatsch, 2013) | No | Yes | Yes | Yes | No | No | Yes | Yes |
| The communication skills used by deaf children and their hearing peers in a question-and-answer game context  (Toe & Paatsch, 2010) | No | Yes | Yes | Yes | No | No | Yes | Yes |
| The development of pragmatic skills in children who are severely and profoundly deaf  (Toe et al., 2013) | No | Yes | Yes | Yes | No | No | Yes | Yes |
| Conversational fluency of children who use cochlear implants  (Tye-Murray, 2003) | No | No | No | Yes | Yes | Yes | Yes | Yes |
| Early intervention parent talk and pragmatic language in children with hearing loss  (Yoshinaga-Itano et al., 2020) | No | No | No | Yes | No | No | No | Yes |
| Pragmatics and peer relationships among deaf hard of hearing and hearing adolescents  (Zaidman-Zait & Most, 2020) | No | Yes | No | Yes | No | Yes | No | Yes |

**Appendix 2**

*Data Extraction Instrument: Studies about Conversation*

| Title & Author/s | Country / Language | Objectives | Participants | Context | Outcomes |
| --- | --- | --- | --- | --- | --- |
| Some trouble with repair: Conversations between children with cochlear implants and hearing peers  (Church et al., 2017) | Australia (English) | To understand differences in pragmatic skills of HoH children with CIs compared with matched TH peers, by using conversation analysis. | 10 DDH children using CIs and 10 TH matched peers (by grade and gender), creating 10 dyads, each recorded for 10 minutes of free conversation.  Small subset of larger cohort discussed here from later cohort (see Paatsch & Toe, 2014). | CA used to look at incidence of interactional troubles in 10-minute conversational samples.  Focus on how children who are HoH interact with peers with TH in mainstream inclusive education settings. Children used spoken language. | Other-initiated repairs (OIRs) that prompt speaker to repeat prior utterance more commonly produced by children with CIs. In some cases, child with CI chose NOT to initiate repair of an error made by their peer. TH peers never did this. |
| What's that you say? Communication breakdowns and their repairs in children who are deaf or hard of hearing  (Fitzpatrick et al., 2020) | Canada (English) | To describe the conversational fluency of HoH children in free conversation with a TH adult. | 14 Canadian HoH children aged 7-12 years, compared with 15 TH children matched by age, all children in conversation with a TH adult – all children had average non-verbal intelligence.  Most HoH children used a HA or HAs. Range of ages at diagnosis from 0 to 84 months. More males than females. | 10-minute conversational sample recorded following protocol described by Miller & Chapman (2012) using SALT software.  Compared number and duration of communication breakdowns, requests for repair, and responses to requests used by child. | HoH children demonstrated some differences, their breakdown and repair behaviors were more like TH peers than previously reported. |
| Social conversational skills development in early implanted children  (Guerzoni et al., 2016) | Italy (Italian) | To understand development of social conversational skills, with a focus on assertiveness and responsiveness (measures of early pragmatic development), in early implanted HoH children with CIs. | 28 HoH children with CIs, average CA 29.5 months (range 20-36 months), average age at implant 13.3 months (range 8-24 months).  Results compared to normative data. | Skills measured using ‘The Social Conversational Skills Rating Scale’ (Girolametto, 1997). | Children with CIs performed within the normal range on measures of assertiveness and responsiveness.  Age at implant activation, and pre-operative residual hearing had a positive impact on assertiveness and responsiveness. Highest scores with youngest implantees, and those with more hearing before implantation.  After 1 year of CI use, children matched their same-aged peers on these measures.  Lexical development strongly correlated with conversational skills, age at diagnosis and maternal education not correlated with social conversational skills. |
| Discourse strategies and the production of prosody by prelingually deaf adolescent cochlear implant users  (Holt et al., 2017) | Australia (English) | To assess the use of discourse strategies and the production of prosody by prelingually deaf adolescent with CIs in a referential communication task. | 8 HoH children CA range from 12-14.2 years, compared with 8 TH peers CA range from 12-14.2 years.  Average age at CI was 2;1 (SD 0.9). | Pairs completed referential communication tasks, directing their partner around a map.  3 Dyad Combinations:  1) HoH as expert / TH as novice,  2) TH as expert / HoH as novice,  3) TH as expert / TH as novice. | CI users preferred using and repeating directives, rather than requesting information (asking questions) to understand the communication partner’s point of view. HoH children. Used more directives and less acknowledgements than TH peers. |
| Speech recognition working memory and conversation in children with cochlear implants  (Ibertsson et al., 2009a) | Sweden (Swedish) | To understand the interaction between speech recognition, working memory and conversation skills in children/adolescents with CIs in conversation with TH peers to see if either of these factors correlated with better or worse conversational skills. | 13 HoH children with CIs, CA range from 141-229 months, average duration of implant use ranges from 50 to 165 months.  Compared with 8 TH children, CA ranged from 9 to 18 years, matched for age and gender. | Structured referential communication task. Measured # of requests for clarification, time to solve task (in turns), and proportion of different types of requests (RIs). | Speech recognition and working memory influenced type and # of requests for clarification.  Children with better working memory used more requests for confirmation of new information, and less requests for confirmation of already given information, compared to those with poorer working memory.  Children with better speech recognition had better outcomes on measures of conversation. |
| Deaf teenagers with cochlear implants in conversation with hearing peers  (Ibertsson et al., 2009b) | Sweden (Swedish) | To understand how HoH children with CIs co-construct dialogue during referential communication tasks with a TH peer. | 8 HoH children with CIs, average CA 189 months (range 141-229 months), implant age not stated, average length of implant use 111 months (range 74-165 months).  Compared to 8 TH children (11-19 years) matched for age and gender. | Structured referential communication task. Measured # of words, # of turns, time to complete task and the # of times that each type of RI was used, firstly for each pair, and secondly for each type of dialogue.  HoH children talking to TH peers, compared with TH peers talking to TH peers. | Children with CIs made more requests for confirmation of new information and less to confirm given information or request elaboration. |
| The pragmatic skills of profoundly deaf children  (Jeanes et al., 2000) | Australia (English) | To understand how HoH children request clarification, respond to requests, and use communication repair strategies when there is a conversation breakdown. | Total of 40 HoH children with profound HL. 20 in oral education and 20 were using signed English AND spoken English. No other identified disabilities. No info on hearing technology, but as published in 2000 likely to be hearing aids.  Within each group of 20 participations there were three pairs of 8-year-olds, three pairs of 11-year-olds, two pairs of 14-year-olds, and two pairs of 17-year-olds.  Each HoH student was paired with a TH student who they knew. | Used a standard referential communication experimental paradigm from the literature. A  series of five tasks to elicit interactive communication between the participant dyads, with each task including six trials.  Specifically looked at how the person in the role of ‘listener’ requested clarification, and responded to requests, and how the person in the role of ‘speaker’ and used repair strategies during breakdowns. | Differences found between groups suggests that profoundly deaf students had difficulty consistently using appropriate, productive pragmatic behaviors in their face-to-face dyadic interactions (compared to TH peers). |
| Development of implanted deaf children's conversational skills  (Le Maner-Idrissi et al., 2010) | France (French) | To investigate difficulties experienced by children who are HoH in acquiring social rules and social skills related to discourse. | 18 HoH children with CIs, CA range from 26–69 months.  Average IA = 41 months (range 26–69 months).  No comparison group. | Assessed pre-implant, and every 6 months post implant for 2 years. Recorded and analyzed 7.5 min conversational samples.  Pre-implant, HoH children primarily using signed French. Post-implant, communication mode changed to almost exclusively oral. | Assertives (descriptions of the world), directives (requests for action), and expressives (expressions of psychological states) significantly increased over time post-implant, with assertives being the most used speech act. |
| Conversations between deaf children and their hearing mothers: pragmatic and dialogic characteristics  (Lederberg & Everhart, 2000) | America (English) | To understand the pragmatic and dialogic characteristics of conversations between HoH children and their mothers and test the assumption that the way mothers respond their HoH child discourages active participation. | Total of n = 20 HoH Caucasian children with severe to profound HL, but not all using spoken language only. Different etiologies, using HAs. Aged between 22 months and 3 years.  Compared with 20 TH children and their mothers.  Matched by gender, maternal education, marital status, maternal employment. | All accessing center-based all day educational program when they were 36 months old. Variety of language programs and language modalities.  Compared HoH child and their TH mother, with TH child and their TH mother. | HoH children less skilled at maintaining topics. Pragmatic function of communication acts was more likely to be unclear, compared to TH children.  HoH children more likely to direct their mothers and less likely to ask questions than TH children.  Some differences in how mothers responded to HoH children but overall, not significant. Maternal interaction style not supported, as the changes mothers made to their response were apparently related to language delay. |
| Oral conversations between hearing-impaired children and their normally hearing peers and teachers  (Lloyd et al., 2001) | UK (English) | To understand how HoH children talking with TH peers and teachers. | 12 HoH children (average CA 8;8 years). | HoH children video recorded while making Lego models with two partners, a TH peer, and then a teacher.  Children served as their own control group. | Frequency of utterances was balanced, but HoH child took more turns in total (verbal and non-verbal) with teachers, than with peers.  With peers, HoH student turns had more utterances and contribution to conversation was proportionally greater in relation to the length of turns and utterances.  With teacher, the teacher talked more and used longer turns and utterances. HoH student turns less utterances, less contribution to conversation proportionally. |
| The use of repair strategies by children with and without hearing impairment  (Most, 2002) | Israel (Hebrew) | To understand how HoH students, with different levels of speech intelligibility, respond to communication breakdown. | 16 HoH students with profound bilateral HL, and language WNL. 9 boys and 7 girls, 8 with good speech intelligibility, and 8 with poor speech intelligibility. Aged 11-17 years (average CA 13.6 years). All wearing HAs bilaterally and reported to be ‘well aided’ with aided PTA 42dBHL.  Compared with 10 TH students aged 11-18 years. | Students described pictures and to responded to a series of three stacked clarification requests (“Huh?,” “What?,” and “I didn’t understand”) presented by the examiner.  HoH good intelligibility with TH adult, vs. HoH poor intelligibility with TH adult, vs. TH child with TH adult. | Repetition was most frequently used strategy by all groups. Significant differences in the use of other repair strategies between groups, and significant differences between choice of strategy across the three clarification request types.  Despite similar levels of age-appropriate expressive language, pragmatic skills differed. |
| Hearing status language modality and young children's communicative and linguistic behavior  (Nicholas & Geers, 2003) | America (English) | To understand differences in pragmatic skills development between three groups: TH children, HoH children communicating orally (OC), and HoH children using simultaneous communication (SC), aged between 1 and 4 years old. | 38 HoH children with severe or profound HL from 1-4 years old using SC, in conversation with their TH primary caregiver. Some used CIs some used HAs.  Date compared with previously published data from two aged-matched groups; 38 HoH children using OC, and 84 TH children. | Analysis on 30 min play sessions between:   1. HoH child (OC) and primary caregiver with TH. 2. 2) HoH child (SC) and primary caregiver with TH. 3. TH child and primary caregiver with TH.   This paper presents SC data (point 2 above) and compares with previously published date on OC and TH dyads (points 1 and 3 above). | All groups showed a significant improvement with age in the communicative behaviors measured; overall trend was toward growth in all age groups (although rate of growth differed).  By age 3 years, a pattern of communicative function use had emerged in all 3 groups.  Patterns for HoH children in SC and OC groups were like each other and to younger TH children, but different to TH age-matched peers. |
| A comparison of pragmatic abilities of children who are deaf or hard of hearing and their hearing peers  (Paatsch & Toe, 2014) | Australia (English) | To understand the pragmatic skills of children who are HoH in free conversation with their hearing peers. | 31 HoH children with CIs or HAs, average CA = 132 months (range = 114–153 months). Hearing loss ranged from mild to profound. Mix of language levels, classified as ‘at/above’ average or ‘below’ average.  62 Children with TH, average CA = 127 months (range 88-151 months), matched for gender and grade. | 10-min free sample recorded and analyzed, looked at # of turns per partner, # of topic initiations per partner, mean length of turns (words).  Also looked a #topics per conversation, # of turns per topic, # of pauses between turns (2 sec or more) and average pause time.  HoH/TH dyads vs. TH/TH dyads with cross over of children in TH groups. | Children with HL took longer turns, asked more questions, made more personal comments, and initiated more topics than TH peers. |
| Assessing children with profound hearing loss and severe language delay: getting a broader picture  (Remine et al., 2003) | Australia (English) | To understand conversation and pragmatic skills HoH children with severe/ profound using CIs and/or HAs. | 6 HoH children with CIs, average CA = 75 months (range 61–92 months). Average IA = 20 months (range 9–29 months).  2 children had binaural HAs, and 4 children had a unilateral CI (in presence of binaural HL).  No comparison group. | Social competence assessed using questionnaire developed by Brown et al. (2000). Looked at social competence for the dimensions of egocentrism, responsiveness, awareness, affiliation, reciprocity, mutuality, and social problem solving.  Observed children in classroom interactions for 1h / day, for 4 weeks to make ratings. | HoH children could recognize turn-taking cues, but most could not give cues to others to respond, or take appropriate and meaningful turns, or allow others to do so.  Conversational attentiveness measure suggests that all HoH children were at least ‘alert’ and looking for communication opportunities.  Pragmatic skill rated as ‘low’, ranging from 2.25 to 3.75 from a maximum rating of 5. |
| Clarification requests in everyday interaction involving children with cochlear implants  (Samuelsson & Lyxell, 2014) | Sweden (Swedish) | To understand requests for clarification in everyday interaction in HoH children with CIs to determine whether task-oriented communication findings are generalizable to naturally occurring conversation. | 7 HoH children aged 3;6 to 6;3 years (average CA 4;4 years), children using CIs. | Video recordings of interaction between child with CI and same aged TH peer. 7 x 30min recordings taken during ordinary play (total of around 4h recording, or 1 x 30 min recording per pair). | Children with CIs use general and specific requests for clarification (mainly general).  Children with better speech intelligibility use less clarification requests (and vice versa).  Everyday interactions are different from experimental studies or assessment of task-oriented interactions, findings can’t be easily translated between interactional settings. |
| You sometimes get more than you ask for': responses in referential communication between children and adolescents with cochlear implant and hearing peers  (Sandgren et al., 2011) | Sweden (Swedish) | To understand responses to requests for clarification in conversations between HoH children/adolescents with CIs and children/adolescents with TH. | 13 HoH children with CIs, CA: 181 months (range = 141–229).  39 TH children, CA 11 to 19 years, matched for age and gender. | Structured sample (barrier game) Codes for the Human Analysis of Transcripts (MacWhinney, 2000). | No significant differences in type or distribution of requests for clarification in referential communication task. |
| Communicative competence of oral deaf children while explaining game rules  (Toe & Paatsch, 2018) | Australia (English) | To compare how 3 groups of upper primary school students use language to teach a peer to play an unfamiliar board game. | 20 HoH children with HL ranging from mild to profound, all using spoken language.  48 TH children.  Total of 68 children, 8;2 to 13;3 years, 33 girls and 35 boys. | All HoH students were enrolled in mainstream schools in Melbourne and used spoken language as the main mode of communication.  Compared groups as follows:   1. 20 dyads of HoH vs. TH   14 dyads of TH vs. TH | All groups could convey game rules and purpose and navigate clarifications.  Differences emerged in the accuracy of the use of referents when instructing their peers how to play the game.  Specific content vocabulary and the need to emphasize new concise information also challenged HoH children. |
| The conversational skills of school-aged children with cochlear implants  (Toe & Paatsch, 2013) | Australia (English) | To understand conversation skills of HoH children with CIs in free conversation with TH peers. | 20 HoH children with CIs, school-aged, average CA 132 months (range 114-153), IA average 29 months (range 13-51 months).  40 TH children, average CA: 123 (range 108–148).  Groups matched by sex, grade. | 10-min free conversation sample, comparing groups as follows:   1. 20 HoH vs. TH dyad 2. 20 TH vs. TH dyads   (with cross over TH group)  Pragmatic skills analyzed in terms of conversational balance, conversational turn types and conversational maintenance. Impact of speech intelligibility also assessed. | Children with CIs asked more questions, initiated more topics, and took longer turns - essentially conversing in a similar way to some deaf adults who tend to take control of the conversation.  Overall, conversations were not problematic, but similarities between school aged HoH children and what’s seen with HoH adults. |
| The communication skills used by deaf children and their hearing peers in a question-and-answer game context  (Toe & Paatsch, 2010) | Australia (English) | To understand the communication skills of HoH children and their TH peers in a question-and-answer game context e.g., a trivia game. | 34 HoH children with HL ranging from mild to profound, using combination of CI and HAs.  34 TH children with TH, matched by gender and grade level. | Dyads were HoH child and a TH peer. Pairs took turns in each role as speaker/listener. Dyads were videotaped and analyzed.  Pairs were compared in terms of their capacity to repeat the question, strategies used to seek information, and accuracy of responses. | TH children could repeat more questions verbatim compared to HoH children.  HoH group required significantly more repetitions, needed more general clarifications, and correctly answered more questions compared with the TH group. |
| The development of pragmatic skills in children who are severely and profoundly deaf  (Toe et al., 2013) | Australia (English) | To understand conversational skills of HoH children in free conversation with their TODs. | 18 HoH children with CIs and HAs (severe to profound HL), average CA not stated but range from 77-169 months.  No comparison group. | 10 min free conversation sample using assessment with custom schema. Recorded conversation between HoH child and TOD.  Cohort from auditory-oral programs at RIDBC in Sydney.  Looked at balance, turns, missed turns, verbal turns, nonverbal turns, child verbal turns by speech act, conversational breakdowns. | Older children had more conversational balance and response complexity. Few questions and breakdowns occurred as children got older. |
| Conversational fluency of children who use cochlear implants  (Tye-Murray, 2003) | America (English) | To assess oral conversational fluency of HoH children using CIs | 18 HoH children with CIs, CA from 8–9 years, IA 37 months (SD 5–64 months).  24 TH children, CA 8 to 9 years matched for gender and age distribution | 10-min free sample DYALOG conversational analysis (Erber, 1996).  Compared groups as follows:   1. HoH Oral vs. TH 2. HoH Sim Com vs. TH   Conversational fluency defined as how smoothly a conversation unfolds, that is, less breakdowns means more fluent. | Children with CIs spent much more time in communication breakdown. Oral and hearing children showed less conversational balance than Sim Com children. |

**Abbreviations:** HA = hearing aid/s, HL = hearing loss, CI = cochlear implant/s, CA = chronological age, HoH = deaf and hard-of-hearing, TH = typical/ly hearing, SD = standard deviation, IA = implant age, TODs = teachers of the deaf, SNHL = sensorineural hearing loss, SN = sensorineural, PTA = pure tone average, SES = socio-economic status, RI = repair initiator / initiations

**Appendix 3**

*Data Extraction Instrument: Studies about Pragmatics*

| Title & Author/s | Country / Language | Objectives | Participants | Context | Outcomes |
| --- | --- | --- | --- | --- | --- |
| A longitudinal study of pragmatic language development in three children with cochlear implants  (Dammeyer, 2013) | Australia (English) | To observe speech intelligibility, auditory performance, turn taking, repair behaviors and gaze (aspects of pragmatics) in HoH children over time. | 3 HoH children with CIs. Average age 60 months (range 57-63 months) at first recording. Average IA = 24 months (range 18–29 months). | Children observed in natural interactions (90 mins of free conversation) 6 times / year for 3 years, then 2 times in the final year (total duration of 4 years). Self-developed schemas were used to assess skills in the observed conversations. | Children improved their speech intelligibility and auditory performance during the 4 years, but they continued to have difficulties with pragmatic language usage. |
| Peer interactions of preschool children with and without hearing loss  (DeLuzio & Girolametto, 2011) | Canada (English) | To understand how children with severe to profound HL develop social interaction skills by investigating how they manage conversational exchanges with TH peers. | 12 HoH children CA range 37-62 months (average CA 49.3 month) with severe to profound HL, 6 using CIs and 6 used bilateral HAs, compared with 12 TH children matched for intelligence, language, speech, and social skill development.  Diagnosis made before 24 months of age. | Observations of Initiation and response skills during 20 minutes of group play. Integrated preschool programs.  Outcome measures included number and type of initiation strategies, number of responses, and length of interactions. Self-developed schema used. | HoH children had poorer speech, language, and social development than TH peers overall, however, there were no significant differences in initiation and response skills between groups.  Having age-appropriate language skills did not ensure successful peer interactions. HoH preschool children were excluded from interactions by their playmates.  Playmates initiated interactions with HoH children less often than with other TH children. TH children ignored initiations from HoH children more often than those of other TH children. |
| The missing link in language development of deaf and hard of hearing children: pragmatic language development  (Goberis et al., 2012) | America (English) | To describe pragmatic development of TH and HoH children. | 126 HoH children from 2-7 years, with CIs, compared with 109 TH age-matched peers from 2-7 years.  CA range from 26–69 months, average IA 41 months (range = 26–69).  Data analyzed by age groups,  18 to 29 months, 30 to 41 months, 42 to 53 months, 54 to 65 months, 66 to 77 months, 78 to 89 months, and 90 months.  Between 30 and 50 participants (HoH and TH) included in each age grouping. | All children had normal cognitive development.  Degree of HL varied; 19.8% of HoH children had mild HL, 24.2% had moderate to moderate-severe HL, 32.9% had severe HL, and 23.1% had profound HL.  Skills measured using 'The Pragmatics Checklist'. (Goberis, 1999). | TH children mastered 44% (20 of 45) of items using ‘complex language’ by CA3, 95.5% (43 of 45) of items by CA, 98% of items by CA5, and 100% of items by CA6.  HoH children mastered 6.6% (3 of 45) of the items with ‘complex language’ by CA6, and 69% (31 of 45) of items by CA7.  HoH children were significantly older than TH peers when they demonstrated items on the assessment with ‘complex language’. |
| Children with hearing impairment and early cochlear implant: A pragmatic assessment  (Hilviu et al., 2021) | Italy (Italian) | To understand the relationship between age at CI, and development of pragmatic skills in HoH children. | 18 HoH children who received CI before CA2, compared with TH peers matched by age. All otherwise typically developing.  Assessments completed for three groups aged 6;11 to 7;11, 8;0 to 8;11, and 9;0 to 9;11 years. | Measured areas of pragmatics including understanding metaphors, implicit meaning, comics, situations using ‘The Pragmatic Language Skills Test’ (APL-Medea) (Lorusso, 2009) which includes measuring perspective taking using ‘The Colors Game’. | HoH children with CI achieved lower scores than TH peers.  HoH children with CIs differed from TH kids in comics, and colors game tasks.  Age at implantation was a moderate but significant predictor of pragmatic performance. |
| Early pragmatics in deaf and hard of hearing infants  (Kelly et al., 2020) | UK (English) | To understand whether HoH infants are more or less likely to engage in gestural and vocal pragmatic behaviors, compared with normative info on 5 types of infant commination known to positively predict later language development (show gestures, give gestures, index finger pointing, communicative vocalizations and early word use). | 8 HoH infants aged 12-18 months old, with  HL ranging from moderate to profound HL. Infants received HAs at between 5 and 15 weeks old. No additional needs, not premature or low birth weight.  Most families used spoken language only (but mixture of spoken and sign-supported).  More male than females in sample.  Compared with 8 TH infants matched for age, gender, and SES. | Observing natural interactions, free play with 25 mins at home was recorded, and then self-developed checklist was administered.  Analyzed the frequency of use of 5 types of infant communication known to positively predict later language development (i.e., show gestures, give gestures, index-finger pointing, communicative vocalizations, and early word use). | Hearing loss had a significant negative effect on how frequently infants engaged in all types of early communication that can predict later language development.  HoH infants are at high risk of delay in the gestural and vocal communicative skills that lay the foundations for later language. Delays both in gestural and vocal domains suggests that it’s not just HL, but interactive experiences that are impacted. |
| The effect of age at time of cochlear implantation on the pragmatic development of the prelingual hearing impaired children  (Khodeir et al., 2021) | Egypt (Arabic) | To understand whether age at CI (pre/post 3 years old) influences pragmatic language development in HoH children. | 60 HoH Arabic speaking children between 4 and 7;4 years, with pre lingual bilateral severe to profound SNHL and ‘normal auditory levels’ by CI aided audiogram.  All children had 6 months post-CI rehab.  30 children implanted before 3 years and 30 implanted after 3 years old. | Egyptian Arabic Pragmatic Language Test (EAPLT) (Khodeir et al., 2017). Scores at 5^th^ %ile interpreted as ‘child has acquired the skill’, while scores at 95^th^ %ile interpreted as 'child has mastered the skill'.  Average CA at diagnosis for PRE 3 years group was 8 months (average age when HAs fitted was 2.3 years), and POST 3 years group was 1.9 years (average when HAs fitted was 4.7 years).  Compared CI before 3 years old, with CI after 3 years old. | HoH children had poor pragmatic skills whether they received CI before 3 years or after 3 years.  Scores of the EAPLT were positively correlated to child’s CA, their language abilities, and duration of language rehabilitation provided. No significant correlation between EAPLT and CA at implantation. |
| Adequate formal language performance in unilateral cochlear implanted children: is it indicative of complete recovery in all linguistic domains? Insights from referential communication  (Mancini et al., 2015) | Italy (Italian) | To understand referential communication skills in HoH children with severe/profound HL. | 31 HoH children with unilateral CIs (in presence of bilateral loss, some had contralateral HA), and language development WNL.  Age at diagnoses ranged from 4 months to 36 months, various etiology. Age at CI ranged from 9 months to 41 months, and IA ranged from 69 months to 127 months. | Pragmatics skills assessed with ‘The Pragmatic Language Skills Test’ (APL-Medea) (Lorusso, 2009) including ‘The Colors Game’ played with their parent.  Game scored on a 3-poin scale, in five areas; description of materials, step rule, joker function, black face function, and how to win. Normative data available. | 83.9% HoH children performed appropriately for their CA.  CI had a positive effect on referential communication, although some CI users continued struggle. |
| Pragmatic abilities of children with hearing loss using cochlear implants or hearing AIDS compared to hearing children  (Most et al., 2010) | Israel (Hebrew) | To characterize pragmatic abilities of HoH children (using HAs or CIs). | 24 HoH children, 13 using HAs, and 11 using CIs. Average CA = 91 months (range = 79–103 months), average IA = 30 months (range = 14–60 months.  Compared with 13 TH children average CA = 88 months (range 68-112 months), matched on language age. | 15-min free sample used to inform completion of The Pragmatic Protocol (Prutting & Kirchner, 1987).  HoH children used spoken language, attended mainstream schools, and accessed communication therapy twice a week.  Results from HoH children using CIs and HAs, compared to results from TH peers. | More inappropriate pragmatic behaviors were used by > 50% of HoH children (n = 10) compared with TH children (n = 2). |
| Pragmatic skills in children with hearing loss: comparison between cochlear implants and hearing aids users  (Rezaei et al., 2021) | Iran (Persian) | To understand whether HoH children with CIs have better pragmatic skills than HoH children with HAs. | 52 children in 3 groups, HoH CI users (n = 16), HoH HA users (n = 16), TH children (n = 20).  Average CA was 6 years old. | Persian version of the Children’s Communication Checklist (Kazemi, 2007). | HoH children had acceptable pragmatic skills compared with TH peers based on parent-report.  Suggests that HoH children at early stages of language development can develop pragmatic skills despite language delay. |
| Linguistic and pragmatic skills in toddlers with cochlear implant  (Rinaldi et al., 2013) | Italy (Italian) | To compare linguistic skills of children implanted by 12 months of age with children implanted between 13 and 26 months of age; and to describe the relationship among lexical, grammar and pragmatic skills. | 12 HoH children with CIs, average CA 29 months (range = 24–34 months), average IA = 15 (range = 9–26).  Compared with normative data | Assessment - Le Abilità Socio- Conversazionali del Bambino (Girolametto, 1997). | Most children with CIs performed below the mean, with many outside the normal range. CI may provide HoH children with opportunity to develop language skills, but difficulties in early social experiences and interaction remains. |
| Study of pragmatic language ability in children with hearing loss  (Shoeib et al., 2016) | Egypt (Arabic) | To examine the pragmatic abilities of HoH children with SNHL with goal to develop intervention program to mitigate effect of early pragmatic difficulties on later academic and social abilities. | 27 HoH children, HL severities including mild (n = 9), moderate (n = 8), moderately severe (n = 5), and severe (n = 5) loss. Average PTA 61.04 (+/1 15.14 dB). Average CA 8.78 years (+/- 2.04 years, range 30-80dB).  All children using bilateral HAs with 'satisfactorily aided responses’. All used spoken language ‘sufficiently well’ to qualify for the study.  Compared with 27 TH age and gender matched peers. | Used Arabic versions of Test of Pragmatic Language, Observational Rating Scale and Pragmatic Profile subsets of the CELF-4 (Alduais, 2012). Also collected standardized language scores.  Children had no other disabilities and did not use sign language. 16 children were in regular classes. | Significantly lower pragmatic abilities in children with HL compared with children with TH.  Male HoH children worse than female HoH children.  Significant correlations between pragmatic variables and degree of HL, speech discrimination ability, and the duration of auditory deprivation. |
| Pragmatic language skills: A comparison of children with cochlear implants and children without hearing loss  (Socher et al., 2019) | Sweden (Swedish) | To compare pragmatic skills of HoH children with CIs to those with TH hearing. | 14 HoH children with CIs from age 5;7 to 8;11 years (preschool, first and second graders). 10 girls, 4 boys, average CA6.77 years (SD = 11.13 months).  Diagnosis made at average age of 11.14 months (SD = 13.84 months), 3 children unilateral CI, 11 bilateral CI. Average age at implantation 24.07 months (SD = 19.55 months).  2 children bilingual (sign language and oral language), 4 children used only oral language, 8 used oral language mainly and signs for support.  Compared with 34 TH children matched by age. | HoH children from special school and hearing clinic in Sweden, TH children from local mainstream school.  The Pragmatics Profile of the CELF-4 (Semel et al., 2003) to assess pragmatics, other assessments used to measure vocabulary, grammar, and other verbal cognitive measures. | Many HoH children with CI show pragmatic language ability like their TH peers.  Significant differences were found on a pragmatic measure connected to theory of mind which has been found to be delayed in deaf and hard of hearing children.  Verbal fluency correlated with all three sub-measures of pragmatic language ability in the Pragmatics Profile (causal direction is unclear). |
| Pragmatic language in deaf and hard of hearing students: correlation with success in general education  (Thagard et al., 2011) | America (English) | To understand the relationship between socio-linguistic pragmatic competence in HoH students, and the student’s degree of loss, communication mode, and their degree of success in general education using self-developed checklists. | 81 HoH children from preschool to grade 8, mixture of spoken and signed language users, range of degree of HL, 1/3 used CIs, 2/3 used HAs. 59 used spoken language and the remaining 22 used signed language.  No comparison group. | Self-developed measures used. | Better socio-pragmatic language skills were highly positively correlated with academic outcomes in HoH children, regardless of communication modality (spoken or signed language). |
| Early intervention parent talk and pragmatic language in children with hearing loss  (Yoshinaga-Itano et al., 2020) | America (English) | To identify variables associated with pragmatic language ability in HoH children. | 124 HoH children with bilateral HL from 4-7 years old, compared to normative data previously reported on ‘mastery’ of skills by TH children.  All used spoken English, 16% also used sign language with spoken language. Most had no additional disabilities, 10% did report additional disabilities. Range of non-verbal IQs, 4% scored >2SDs below average.  Degree of HL ranged from mild to profound, all used HAs or CIs. 51% were male, 84% primarily spoken language. | Pragmatic skills evaluated annually using ‘The Pragmatics Checklist’ (Goberis, 1999).  Cohort of children in Colorado. | HoH children who met 1-3-6 guidelines (Joint Committee on Infant Hearing, 2019) scored higher on this measure than HoH children who did not meet 1-3-6 guidelines (Joint Committee on Infant Hearing, 2019).  HoH children who were a) exposed to more parent talk, b) had higher non-verbal intelligence, c) lesser degree of HL and d) whose mothers were more educated, also did better. |
| Pragmatics and peer relationships among deaf hard of hearing and hearing adolescents  (Zaidman-Zait & Most, 2020) | Israel (Hebrew) | 1. To understand differences in pragmatic abilities and peer relationships between HoH adolescents and TH peers. 2. To explore the contribution of pragmatic skills and speech (i.e., articulation and intelligibility) to social aspects of school functioning. | 33 HoH adolescents with moderate to profound HL from grades 7-11 (57% male, average CA 14.83 years SD 1.12), compared with 34 TH adolescents (53% boys, average CA 14.86 years, SD 1.18) also from grades 7-11.  62% used HAs, and 38% CIs. Overall, 86.7 were bilaterally aided (remainder unilaterally aided). | Used teacher reports on ‘Strengths and Difficulties Questionnaire’ (SDQ) (Goodman, 1997) to look at prosocial behaviors and peer relationships, and The Children’s Communication Checklist-2 (CCC-2) (Bishop, 2003) to assess pragmatic language. Also gathered self-reported info on supportiveness of peer relationship and school engagement. | HoH adolescents had more difficulty interacting with peers than did their TH peers. HoH adolescents demonstrated more pragmatic and speech difficulties than hearing peers, and higher levels of peer relationship problems and less prosocial behavior that their TH peers.  Positive correlation between pragmatic abilities and success in peer relationships and more prosocial behaviors for both TH and HoH participants.  Better pragmatic skills positively correlated with adolescents’ perceptions of peer support, and better speech intelligibility associated with higher levels of school engagement. |

**Abbreviations:** HA = hearing aid/s, HL = hearing loss, CI = cochlear implant/s, CA = chronological age, HoH = deaf and hard-of-hearing, TH = typical/ly hearing, SD = standard deviation, IA = implant age, TODs = teachers of the deaf, SNHL = sensorineural hearing loss, SN = sensorineural, PTA = pure tone average, SES = socio-economic status, RI = repair initiator / initiations

**Appendix 4**

*Ovid MEDLINE Search Strategy*

| Set | Search Statement |
| --- | --- |
| 1. | adolescent/ or child/ or child, preschool/ or infant/ |
| 2. | child*.mp. |
| 3. | p?ediatric*.mp. |
| 4. | infant*.mp. |
| 5. | adolescent*.mp. |
| 6. | school age*.mp. |
| 7. | preschool*.mp. |
| 8. | preschool age*.mp. |
| 9. | 1 or 2 or 3 or 4 or 5 or 6 or 7 or 8 |
| 10. | communication/ or language/ or nonverbal communication/ or verbal behavior/ or speech/ |
| 11. | child development/ or language development/ |
| 12. | communicat*.mp. |
| 13. | (listen and speak).mp. [mp=title, abstract, original title, name of substance word, subject heading word, floating sub-heading word, keyword heading word, organism supplementary concept word, protocol supplementary concept word, rare disease supplementary concept word, unique identifier, synonyms] |
| 14. | talk*.mp. |
| 15. | speak*.mp. |
| 16. | oral communication.mp. |
| 17. | oral language.mp. |
| 18. | speech.mp. |
| 19. | spoken language.mp. |
| 20. | language.mp. |
| 21. | verbal.mp. |
| 22. | 10 or 11 or 12 or 13 or 14 or 15 or 16 or 17 or 18 or 19 or 20 or 21 |
| 23. | Hearing Aids/ or Cochlear Implants/ |
| 24. | hearing aid*.mp. |
| 25. | hearing device*.mp. |
| 26. | cochlear implant*.mp. |
| 27. | 23 or 24 or 25 or 26 |
| 28. | hearing loss*.mp. |
| 29. | hearing impair*.mp. |
| 30. | deaf.mp. |
| 31. | hard of hearing.mp. |
| 32. | congenital.mp. |
| 33. | pre$lingual.mp. |
| 34. | permanent.mp. |
| 35. | bilateral.mp. |
| 36. | from birth.mp. |
| 37. | born with.mp. |
| 38. | 28 or 29 or 30 or 31 or 32 or 33 or 34 or 35 or 36 or 37 |
| 39. | convers*.mp. |
| 40. | pragmatic*.mp. |
| 41. | social language.mp. |
| 42. | 39 or 40 or 41 |
| 43. | 9 and 22 and 27 and 38 and 42 |
| 44. | limit 43 to (English language and yr=”2000-2022”) |
